# The association of lipid metabolism with bone metabolism and the role of human traits: a Mendelian randomization study

**DOI:** 10.3389/fendo.2023.1271942

**Published:** 2023-12-06

**Authors:** Jian Kang, Shuangli Zhao, Xize Wu, Can Wang, Zongkun Jiang, Shixuan Wang

**Affiliations:** ^1^ Graduate School, Liaoning University of Traditional Chinese Medicine, Shenyang, China; ^2^ Orthopedics and Traumatology, The Second Hospital of Liaoning University of Chinese Medicine, Shenyang, China; ^3^ Department of Critical Care Medicine, Nantong Hospital of Traditional Chinese Medicine, Nantong, China; ^4^ Clinical College, Jinzhou Medical University, Jinzhou, China

**Keywords:** lipid metabolism, bone metabolism, bone mineral density, human traits, Mendelian randomization

## Abstract

**Background:**

The impact of lipid metabolism on bone metabolism remains controversial, and the extent to which human traits mediate the effects of lipid metabolism on bone metabolism remains unclear.

**Objective:**

This study utilized mendelian randomization to investigate the effects of blood lipids on bone mineral density (BMD) at various skeletal sites and examined the mediating role of human traits in this process.

**Methods:**

We leveraged genetic data from large-scale genome-wide association studies on blood lipids (n=1,320,016), forearm bone mineral density (FA-BMD) (n=10,805), lumbar spine bone mineral density (LS-BMD) (n=44,731), and femoral neck bone mineral density (FN-BMD) (n=49,988) to infer causal relationships between lipid and bone metabolism. The coefficient product method was employed to calculate the indirect effects of human traits and the proportion of mediating effects.

**Results:**

The results showed that a 1 standard deviation(SD) increase in HDL-C, LDL-C and TC was associated with a decrease in LS-BMD of 0.039 g/cm^2^, 0.045 g/cm^2^ and 0.054 g/cm^2^, respectively. The proportion of mediating effects of systolic blood pressure (SBP) on HDL-C to LS-BMD was 3.17%, but suppression effects occurred in the causal relationship of LDL-C and TC to LS-BMD. Additionally, the proportion of mediating effects of hand grip strength (HGS) on the TC to LS-BMD pathway were 6.90% and 4.60% for the left and right hands, respectively.

**Conclusion:**

In conclusion, a negative causal relationship was established between lipid metabolism and bone metabolism. Our results indicated that SBP and HGS served as mediators for the effects of lipid metabolism on bone metabolism.

## 1 Introduction

Bone metabolism is a continuous process of bone formation and bone resorption, commonly known as “bone remodelling” (1). “Bone homeostasis” refers to the state of maintaining a delicate balance in bone metabolism, with the amount of bone formed by osteoblasts equaling the amount of old bone absorbed by osteoclasts (2). Disrupting bone metabolism can lead to various metabolic bone diseases, the most common of which is osteoporosis. According to the reports, the global incidence of osteoporosis was approximately 18.3%, and among the elderly population, it has reached about 21.7% (3, 4). With the changing global population structure, the incidence of osteoporosis is steadily increasing year by year, making it a significant public health concern worldwide (5).

Lipid metabolism is the process of synthesis, degradation and transport of lipids in various tissues of the body and plays a crucial role as a regulatory messenger in systemic metabolism (6, 7). It is closely related to bone metabolism, and lipid metabolism disorders can directly affect bone formation and resorption, thereby affecting BMD and strength (8, 9).Despite the increasing number of studies on the relationship between lipid metabolism and bone metabolism, the results are still inconsistent. Several studies have reported that BMD in postmenopausal women is negatively correlated with total cholesterol (TC), triglycerides (TG), low-density lipoprotein cholesterol (LDL-C), and high-density lipoprotein cholesterol (HDL-C) (10–13). Conversely, several other studies have shown that TC, TG, LDL-C and HDL-C are positive or nonsignificantly associated with BMD (14, 15). We speculate that the reasons behind these counterintuitive research findings may be attributed to the influence of sample size and confounding factors on the results. Therefore, further exploration of the field of lipid metabolism and bone metabolism is warranted to gain a deeper understanding of the relationship between the two.

Mendelian Randomization (MR) is an effective method that utilizes genetic variation as instrumental variables (IVs) to investigate the causal relationship between exposure and outcome phenotypes (16). By leveraging the publicly available results of large-scale genome-wide association studies (GWAS), MR allows the inference of a causal relationship between an exposure risk factor and a disease outcome, effectively addressing confounding biases encountered in traditional epidemiological studies. MR analysis necessitates a large sample size, and two-sample MR analysis enhances the sample size and statistical power of MR (17). Multivariate MR(MVMR) serves as a significant extension of traditional MR, enabling the causal effects of multiple exposures on an outcome to be assessed (18).

This study investigated the causal associations between lipids and bone metabolism through a two-sample MR approach. Additionally, it explored the role of human traits in the pathways linking lipid and bone metabolism, making a valuable contribution to the field of metabolism.

## 2 Materials and methods

### 2.1 Research design

This study examined the causal associations between four lipid levels (HDL-C, LDL-C, TC, and TG) and BMD at three specific sites: forearm (FA-BMD), lumbar spine (LS-BMD), and femoral neck (FN-BMD). Firstly, a two-sample univariate MR (UVMR) was employed to assess the causal relationships between lipid levels and BMD at each site. If a significant correlation was found between any lipid level and BMD at any site, additional research was conducted to determine whether lipid levels indirectly influenced BMD through mediators. In the second step, potential mediators were identified based on predefined criteria, and a MVMR was performed to evaluate the direct effect of lipid exposure on BMD after adjusting for these mediators. The study calculated the indirect effects and proportion of mediation. The coefficient product method was applied for estimating indirect effects and proportion of mediation when the direct effect of lipid exposure on BMD decreased following mediator adjustment.

### 2.2 Genetic association of bone metabolism

BMD is widely recognized as an effective indicator for assessing bone metabolism (19). Our study sourced genetic variables associated with BMD were derived from a comprehensive summary of the largest publicly available GWAS meta-analysis conducted by the Osteoporosis Consortium, which focused on individuals of European ethnicity (GEFOS, http://www.gefos.org/?q=content/data-release-2015; 20). The datasets used in our analysis encompass three GWAS datasets with different derived Dual-energy X-ray absorptiometry (DXA) traits, and statistical information was aggregated for FA-BMD (n=10,805), LS-BMD (n=44,731), and FN-BMD (n=49,988). The original GWAS researchers tested the cumulative effects of individual variants with Minor Allele Frequency (MAF) greater than 0.5% on lumbar spine, femoral neck, and forearm BMD. Adjustments were made for gender, age, and body mass index (BMI), and the data underwent weighting and standardization to achieve a mean of 0 and a standard deviation of 1.

### 2.3 Genetic association of lipid metabolism

In order to identify genetic variables associated with lipid mass spectrometry, we utilized data from the Global Lipids Genetics Consortium (GLGC, https://csg.sph.umich.edu/willer/public/glgc-lipids2021/). This dataset presents information on HDL-C, LDL-C, TC and TG levels of individuals with European ancestry (n=1,320,016) (21).The GWAS project researchers made adjustments for age, age^2^, sex, principal components, and any necessary study-specific covariates. Triglyceride levels underwent natural logarithmic transformation to generate residuals, which were subsequently inverse-normalised. Pre-medication levels of individuals taking cholesterol-lowering drugs were approximated by dividing LDL-C values by 0.7 and TC values by 0.8. Residual-based association analyses for most cohorts were performed using linear mixed-model methods in rvtest or similar software (including BOLT-LMM).

### 2.4 Selection of mediators and genetic associations

Based on previous research and routine medical examination program, we identified and included 10 potential mediators that are associated with human body traits. These mediators comprised BMI, waist-to-hip ratio (WHR), hip circumference (HC), waist circumference (WC), fasting glucose, systolic blood pressure (SBP), diastolic blood pressure (DBP), pulse rate (PR), left-hand grip strength (L-HGS), and right-hand grip strength (R-HGS), which are hypothesised to play a role in mediating the relationship between lipid and bone metabolism. To address any potential biases arising from sample overlap, we selected data from multiple consortia involved in GWAS as the source of these mediators. Initially, our mediator selection process involved two criteria: establishing a causal relationship between the exposure and the mediator, and ensuring a consistent causal relationship between the mediator and the outcome.

### 2.5 Selection of instrumental variables

The three assumptions of IVs in MR analysis are as follow: (1) Strong correlation with the exposure variable; (2) Independence from confounding factors; (3) The IVs affect the outcome through the exposure variable rather than through other means. Based on the three underlying assumptions, we established a genome-wide significance threshold of p*<*5×10^−8^ for screening single nucleotide polymorphisms (SNPs) associated with exposure variables. One potential problem arising from linkage disequilibrium (LD) is the impact on SNPs used to capture causal changes, as they may be influenced by other confounding factors, thereby violating the second or third IVs hypothesis. To mitigate the adverse effects of LD, we used clustering (r^2^ *<*0.001, kb=10000). To assess the strength of the selected SNPs and to minimize weak instrumental bias, we calculated the F-statistic. SNPs were considered as non-weak instruments if their F-statistic was >10 and their MAF was*>*0.01. This stringent criterion ensured that the results of MR analysis were not unduly influenced by weak instrument bias.

To address potential bias due to reverse causality (in which SNPs are more strongly correlated with outcomes than with exposures), we used the Steiger-Flering method to filter SNPs. In addition, we employed the MR-PRESSO (Mendelian randomized multivariate residual sums and outliers) test to identify multi-instrument pooled MR tests at the level of SNPs potential outliers with pleiotropic effects.

### 2.6 Subgroup MR analysis

In order to investigate the gender-specific aspects of lipid and bone metabolism, we performed a subgroup analysis stratified by gender to observe the differences in the causal relationships between lipid and bone metabolism in male and female populations.

### 2.7 Reverse MR analysis

We performed a reverse UVMR analysis to assess whether specific site BMD, which had a causal relationship with lipid metabolism in the forward UVMR analysis, had a similar effect on lipid metabolism.

### 2.8 MR analysis

We performed UVMR to assess the overall causal relationships between HDL-C, LDL-C, TC and TG levels and FA-BMD, LS-BMD and FN-BMD. In the UVMR analyses, inverse variance weighting (IVW) was used as the primary method, and a random-effects meta-analysis was used to combine the Wald ratio estimates for each SNP into a single causal estimate for each exposure variable.

In the UVMR analysis, the weighted median, MR Egger and MR PRESSO methods were used to validate the robustness of the IVW results. The weighted median method provided consistent estimates under the assumption that more than 50% of the information was derived from valid instrumental variables. The MR Egger method evaluated if genetic variation had a directional pleiotropic impact on the results with a mean effect different from zero. The MR PRESSO method was employed to test for potential horizontal pleiotropy of peripheral SNPs and assessed the impact of excluding these outliers on the effect of causal estimates. This assumes that the largest candidate set of instruments with similar estimates represents the valid set of instruments. To identify polymorphism, we utilised MR Egger’s intercept, which may indicate a possible violation of the instrumental variable assumption of two-sample MR. In addition, we used the Q’ heterogeneity statistic to assess heterogeneity among instruments.

In the UVMR analyses, we employed Bonferroni correction was used for multiple comparisons with a significance threshold of 0.05/4 = 0.0125. Consequently, we considered exposure-outcome associations with a p-value less than 0.0125 to have a causal effect. We conducted all MR analyses with R software (version 4.0.2; R Foundation for Statistical Computing, Vienna, Austria) and the R packages “TwoSampleMR”, “MendelianRandomization”, “MRPRESSO” and “MVMR”.

### 2.9 Calculation of intermediary effects

**Figure 1:**
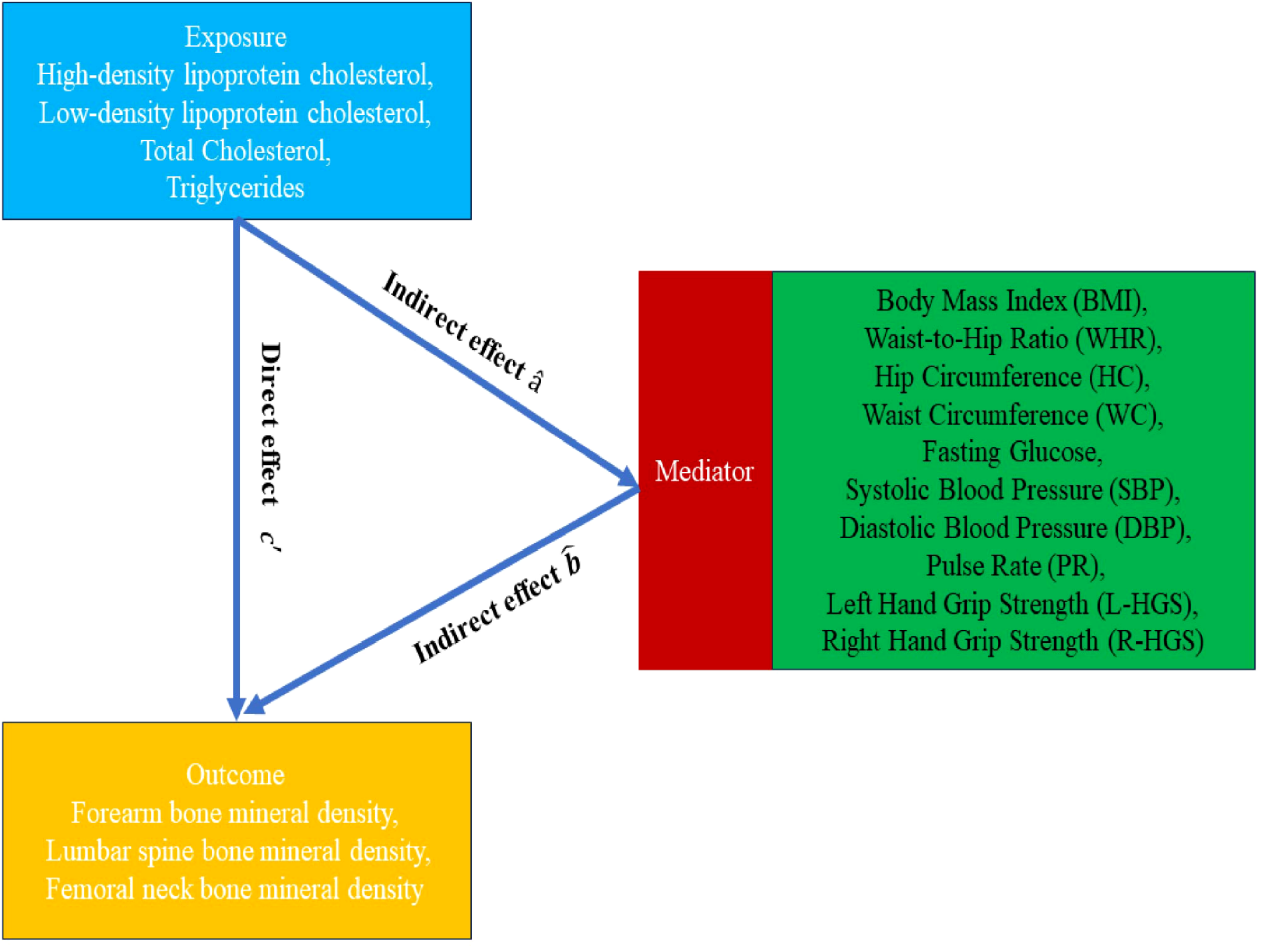
Mendelian randomization flowchart.


If the MVMR analysis indicates that the direct effect of the exposure on the outcome becomes non-significant after adjusting for mediators, the coefficient product method is applied. This method calculates the indirect effect, the standard error of the indirect effect $SE\left(\hat{a}\hat{b}\right)$ and the confidence interval using the delta method. The calculations are performed according to the following formula (22) (**Figure 1**):

(1) $$SE\left(\hat{a}\hat{b}\right)\;=\sqrt{{\left(aSE\left(\hat{b}\;\right)\right)}^{2}\;+\;{\left(bSE\left(\hat{a}\right)\right)}^{2}\;+\;SE{\left(\hat{a}\right)}^{2}\;SE{\left(\hat{b}\;\right)}^{2}\;}$$

(2) $$95\%CI\;=\;\hat{a}\hat{b}\;\pm \;1.96SE\left(\hat{a}\hat{b}\right)\;$$

## 3 Results

### 3.1 Effect of exposure phenotype on outcome

**Figure 2:**
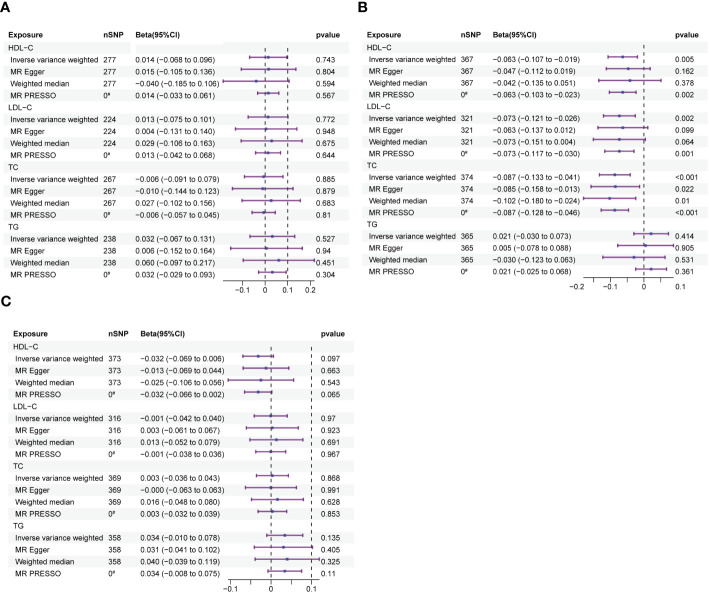
The forest plots depict the correlation between exposure to HDL-C, LDL-C, TC, and TG and their impact on FA-BMD, LS-BMD, and FN-BMD. **(A)** Forest plot of exposure to FA-BMD. **(B)** Forest plot of exposure to LS-BMD. **(C)** Forest plot of exposure to FN-BMD. Forest plots contain nSNP, Beta95CI, and pvalue for the associations of all studies in the analysis. ^#^ for outliers.

Exposures, mediators and outcomes in this study were selected from different consortiums in order to reduce bias in the results due to sample overlap (**Table 1**). In the UVMR investigation of the relationship between the exposure phenotypes and outcome, we selected SNPs with an F-statistic greater than 10 to ensure viability for analysis. To improve the validity of our results, we meticulously excluded any influence from reverse associations or outliers during the analysis process. We discovered a negative correlation between HDL-C, LDL-C, TC and LS-BMD, indicating a potential association between these phenotypes. Despite considering multiple comparisons, the primary IVW analysis consistently produced significant results for all three exposure phenotypes, reinforcing our findings credibility. Although the MR Egger and Weighted Median analyses for HDL-C to LS-BMD, LDL-C to LS-BMD, and the MR Egger analysis for TC to LS-BMD did not reach statistical significance, it is worth noting that the direction of their effects was consistent with the IVW analysis. Furthermore, the IVW results were verified by the MR PRESSO test, thereby confirming their robustness. The beta values for HDL-C and LS-BMD was -0.063 (95% CI: -0.107 to -0.019; p=0.006), for LDL-C and LS-BMD was -0.073 (95% CI: -0.121 to -0.026; p=0.002), and for TC and LS-BMD was -0.087 (95% CI: -0.133 to -0.041; p*<*0.001), as depicted in the results (**Figure 2**). There was no heterogeneity detected, as all p-values were greater than 0.05. In addition, all p-values were greater than 0.05 in the MR-Egger intercept tests, indicating no presence of horizontal pleiotropy (**Table 2**).

**Table 1:** Data sources for the GWAS of the exposure, outcome and mediator are provided.

Phenotype	GWAS ID	No. of SNPs	Consortium	Sample size	Year	Pubmed ID
HDL-C	NA	45150908	GLGC	1320016	2021	36575460
LDL-C	NA	47006483	GLGC	1320016	2021	36575460
TC	NA	46513217	GLGC	1320016	2021	36575460
TG	NA	47196264	GLGC	1320016	2021	36575460
FA-BMD	NA	9723983	GEFOS	49988	2015	26367794
LS-BMD	NA	9726054	GEFOS	44731	2015	26367794
FN-BMD	NA	9291932	GEFOS	10805	2015	26367794
Systolic blood pressure	ieu-b-38	7088083	ICBP-GWAS	757601	2018	30224653
Diastolic blood pressure	ieu-b-39	7160619	ICBP-GWAS	757601	2018	30224653
Pulse rate	ukb-b-15892	9851867	MRC-IEU	151546	2018	NA
Hip circumference	ieu-a-49	2559739	GIANT	213038	2015	25673412
Waist circumference	ieu-a-61	2565408	GIANT	232101	2015	25673412
Waist-to-hip ratio	ieu-a-79	2542432	GIANT	210082	2015	25673412
Body mass index	ieu-b-40	2336260	GIANT	681275	2018	30124842
Hand grip strength (left)	ukb-a-374	10894596	Neale Lab	335821	2017	NA
Hand grip strength (right)	ukb-a-379	10894596	Neale Lab	335842	2017	NA
Fasting glucose	ieu-b-113	2625495	MAGIC	58074	2012	22581228

NA in the GWAS ID column means that the dataset is not included in the MRCIEU database, so no GWAS ID is provided.NA in the PUBMED ID column means that the dataset has no relevant papers published, so no PUBMED ID is available.

**Table 2:** Results of tests for heterogeneity and pleiotropy tests for exposure to outcomes.

	FA-BMD			p	Q	LS-BMD				FN-BMD		
	Q	P	Intercept			p	Intercept	p	Q	p	Intercept	p
HDL-C	299.403	0.998	-6.62E-04	0.368	302.000	0.994	-5.67E-04	0.505	90.162	1	-6.05E-05	0.972
LDL-C	258.329	0.991	-1.44E-04	0.876	268.997	0.982	-3.74E-04	0.721	87.049	1	3.48E-04	0.872
TC	293.922	0.998	1.27E-04	0.883	294.714	0.999	-5.18E-04	0.957	95.418	1	1.55E-04	0.937
TG	311.900	0.959	9.15E-04	0.910	290.672	0.998	4.57E-04	0.623	85.610	1	8.28E-04	0.681

### 3.2 Subgroup MR analysis

In the subgroup analysis of HDL-C, LDL-C and TC data by gender, we observed that in the male group, the The beta values for was -0.063 (95% CI: -0.107 to -0.019; p=0. 005), for LDL-C with LS-BMD was -0.052 (95% CI: -0.101 to -0.004; p=0.034), and for TC with LS-BMD was -0.043 (95% CI: -0.087 to 0.001; p=0.055). In the female group, the beta value for HDL-C with LS-BMD was -0.063 (95% CI: -0.104 to -0.021; p= 0.003), for LDL-C with LS-BMD was -0.044 (95% CI: -0.085 to -0.003; p= 0.036), and for TC with LS-BMD was -0.046 (95% CI: -0.086 to -0.005; p= 0.026), as depicted in the results (**Table 3**). Both tests for heterogeneity and horizontal pleiotropy yielded p-values greater than 0.05, indicating the absence of heterogeneity and horizontal pleiotropy (**Table 4**).

**Table 3:** Results of MR analyses of gender-stratified subgroups.

Gender Exposure	Male			SE	95%CI	Female				95%CI	p
	Method	nSNP	Beta			p	nSNP	Beta	SE		
HDL-C	Inverse variance weighted MR Egger Weighted median	367 367 367	-0.063 -0.047 -0.042	0.022 0.033 0.049	-0.107 to -0.019 -0.112 to 0.019 -0.137 to 0.054	0.005 0.162 0.391	283 283 283	-0.063 -0.05 -0.051	0.021 0.035 0.038	-0.104 to -0.021 -0.118 to 0.018 -0.124 to 0.023	0.003 0.149 0.176
	MR PRESSO	0#	-0.063	0.02	-0.103 to -0.023	0.002	0#	-0.063	0.021	-0.104 to -0.021	0.003
LDL-C	Inverse variance weighted MR Egger Weighted median	230 230 230	-0.052 -0.04 -0.066	0.025 0.039 0.041	-0.101 to -0.004 -0.116 to 0.037 -0.146 to 0.015	0.034 0.308 0.111	251 251 251	-0.044 -0.013 -0.02	0.021 0.031 0.034	-0.085 to -0.003 -0.074 to 0.048 -0.088 to 0.048	0.036 0.673 0.562
	MR PRESSO	0#	-0.052	0.025	-0.101 to -0.004	0.035	0#	-0.044	0.021	-0.085 to -0.003	0.037
TC	Inverse variance weighted MR Egger Weighted median	277 277 277	-0.043 -0.018 -0.041	0.022 0.035 0.039	-0.087 to 0.001 -0.087 to 0.052 -0.119 to 0.036	0.055 0.619 0.297	279 279 279	-0.046 -0.038 -0.011	0.02 0.032 0.034	-0.086 to -0.005 -0.1 to 0.025 -0.079 to 0.056	0.026 0.238 0.743
	MR PRESSO	0#	-0.043	0.022	-0.087 to 0.001	0.055	0#	-0.046	0.02	-0.085 to -0.006	0.026

**Table 4:** Results of tests for heterogeneity and pleiotropy in subgroup MR analysis.

Gender	Male				Female			
	Heterogeneity		Pleiotropy		Heterogeneity		Pleiotropy	
	Q	P	Intercept	p	Q	P	Intercept	p
HDL-C	301.556	0.993	-0.001	0.505	282.64	0.461	<-0.001	0.652
LDL-C	234.044	0.378	-0.001	0.676	256.82	0.353	-0.001	0.186
TC	273.263	0.518	-0.001	0.355	274.474	0.532	<-0.001	0.746

### 3.3 Reverse MR analysis

**Figure 3:**
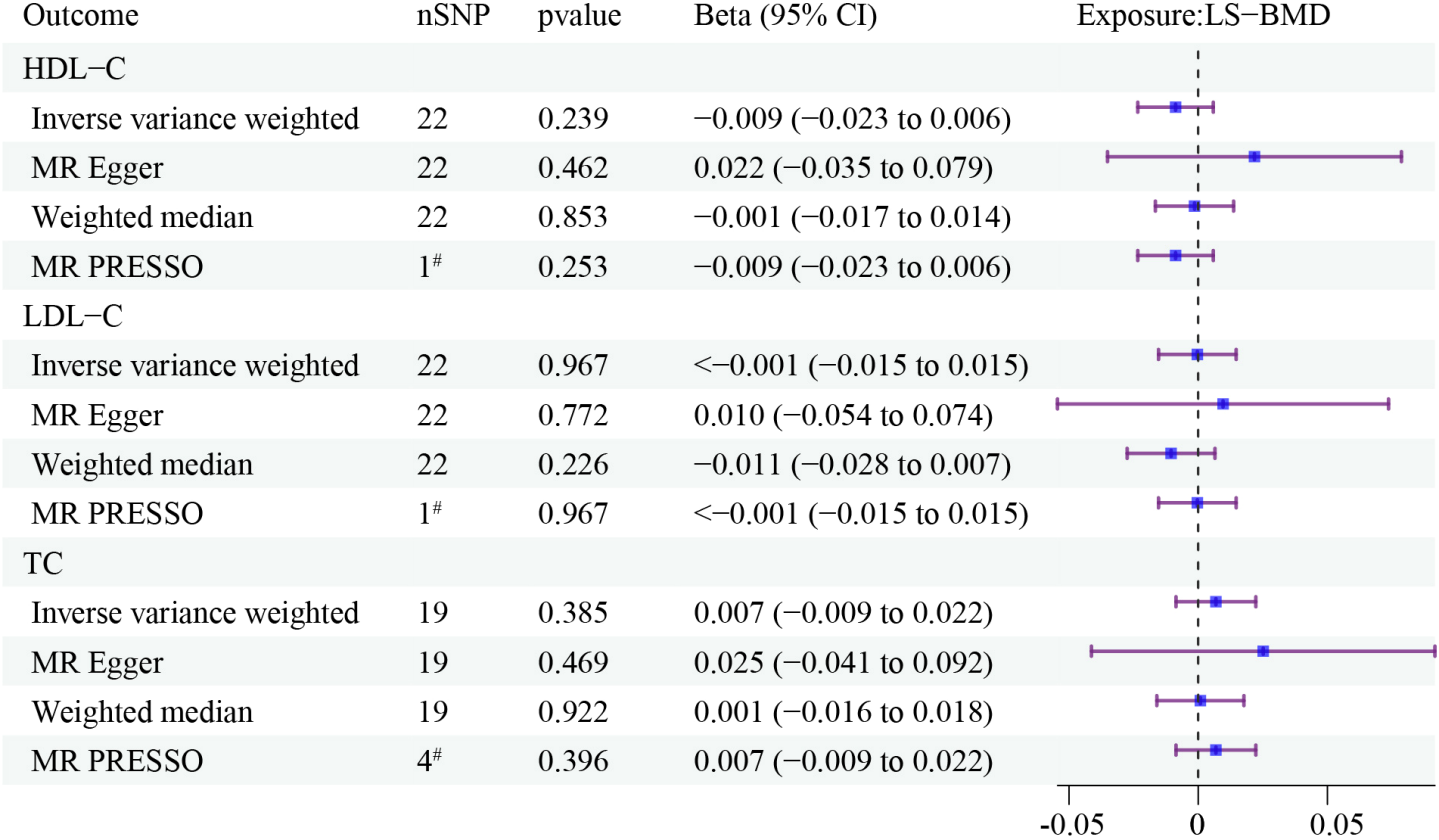
The forest plots depict the correlation between LS-BMD to HDL-C, LDL-C, and TC. Forest plots contain nSNP, Beta95CI, and pvalue for the associations of all studies in the analysis. ^#^ for outliers.

Reverse MR analysis showed that there was no causal association between LS-BMD and HDL-C, LDL-C and TC (**Figure 3**). The results obtained after conducting Cochran’s Q test indicated that there is heterogeneity in the reverse MR analysis. However, no horizontal polytropy was found in the MR Egger test (**Table 5**).

**Table 5:** Results of tests for heterogeneity and pleiotropy in reverse MR analysis.

Outcome	Heterogeneity		Pleiotropy	
	Q	P	Intercept	p
HDL-C	45.274	0.001	-0.002	0.289
LDL-C	49.527	0	-0.001	0.758
TC	41.38	0.001	-0.001	0.586

### 3.4 Effects of exposure phenotypes on mediators

**Figure 4:**
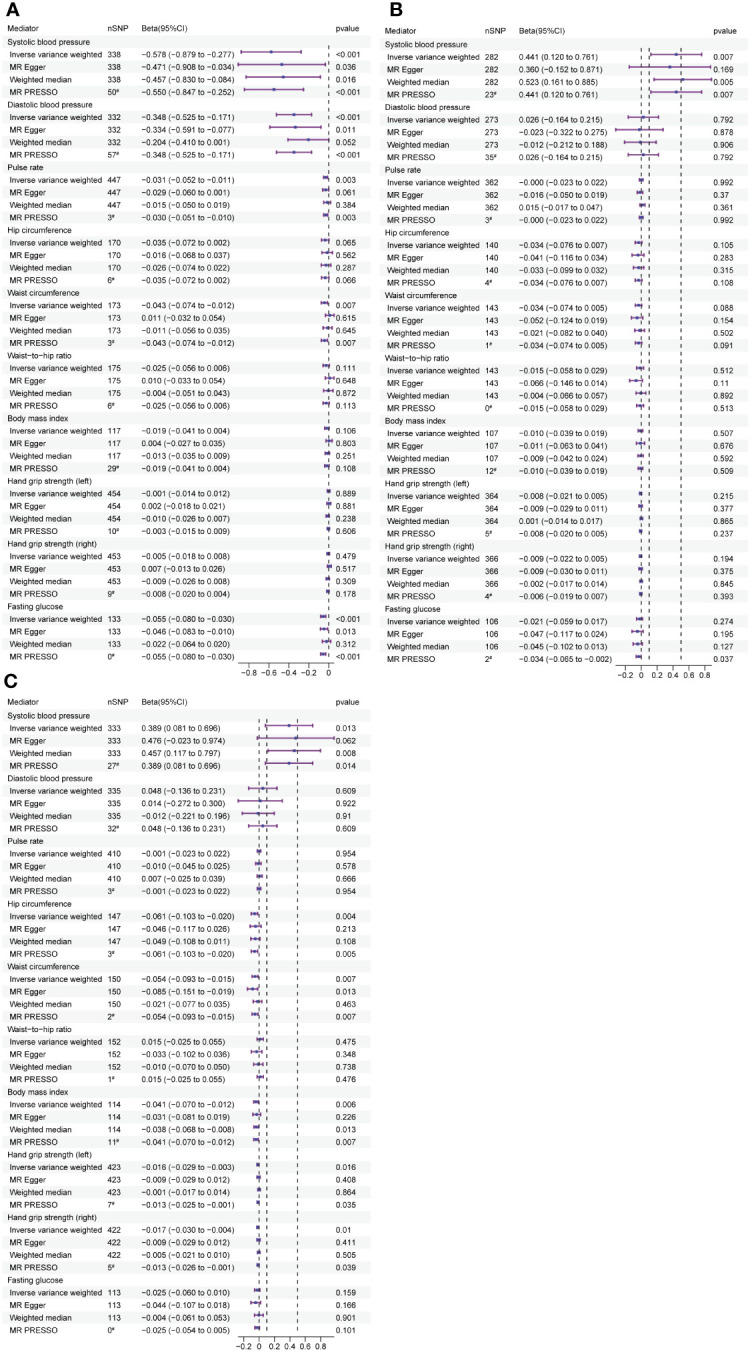
The forest plots demonstrate the relationship between HDL-C, LDL-C, TC and TG exposure and mediator, estimated by UVMR. **(A)** Forest plot of HDL-C to mediator. **(B)** Forest plot of LDL-C to mediator. **(C)** Forest plot of TC tomediator. Forest plots contain nSNP, Beta95CI, and pvalue for the associations of all studies in the analysis. ^#^ for outliers.

We used UVMR to assess the effects of HDL-C, LDL-C, and TC on 10 candidate mediators (**Figure 4**). The results are as follows: (1) HDL-C was negatively associated with SBP(beta: -0.578, 95% CI: -0.879 to -0.277, p ¡ 0.001); (2) LDL-C was positively associated with SBP (beta: 0.441, 95% CI: 0.12 to -0.761, p = 0.007); (3) TC was positively associated with SBP (beta: 0.389, 95% CI: 0.081 to 0.696, p = 0.013), negatively associated with L-HGS (beta: -0.016, 95% CI: -0.029 to -0.003, p = 0.016), and positively associated with R-HGS (beta: -0.017, 95% CI: -0.03 to -0.004, p = 0.010). Cochran’s Q test indicated heterogeneity in the effects of HDL-C, LDL-C, and TC on all mediators, while the MR-Egger intercept test showed no horizontal pleiotropy (**Table 6**).

**Table 6:** Results of tests for heterogeneity and pleiotropy in exposure (HDL-C, LDL-C and TC) to mediators.

	HDL-C				LDL-C				TC			
	Heterogeneity		Pleiotropy		Heterogeneity		Pleiotropy		Heterogeneity		Pleiotropy	
	Q	p	Intercept	p	Q	p	Intercept	p	Q	p	Intercept	p
SBP	1108.588	5.20E-83	-0.004	0.510	823.663	1.05E-54	0.003	0.691	918.049	2.04E-56	-0.003	0.664
PR	610.292	2.94E-07	-5.88E-05	0.873	516.224	1.42E-07	5.38E-04	0.242	587.284	6.50E-08	2.91E-04	0.501
HC	294.370	7.81E-09	-7.19E-04	0.312	163.089	7.95E-02	1.84E-04	0.828	176.940	4.14E-02	-4.10E-04	0.601
WC	245.613	1.93E-03	-2.08E-03	6.28E-04	167.892	6.8E-02	4.78E-04	0.556	173.099	8.61E-02	8.28E-04	0.256
WHR	230.451	2.68E-03	-0.001	0.027	209.770	1.89E-04	0.001	0.138	194.717	9.52E-03	0.001	0.098
BMI	341.203	5.00E-24	-9.10E-04	0.041	292.189	2.48E-19	3.01E-05	0.959	300.112	6.83E-19	-2.58E-04	0.626
L-HGS	1122.595	1.32E-58	-7.83E-05	0.743	752.171	2.2E-29	2.96E-05	0.913	870.828	2.14E-33	-2.28E-04	0.362
R-HGS	1104.712	1.70E-56	-3.58E-04	0.131	786.854	4.80E-33	1.82E-05	0.947	880.115	1.31E-34	-2.81E-04	0.284
Fasting glucose	134.192	0.431	-3.15E-05	0.517	135.433	0.024	6.93E-04	0.397	81.158	0.988	4.86E-04	0.464

### 3.5 Effects of mediators on outcome

**Figure 5:**
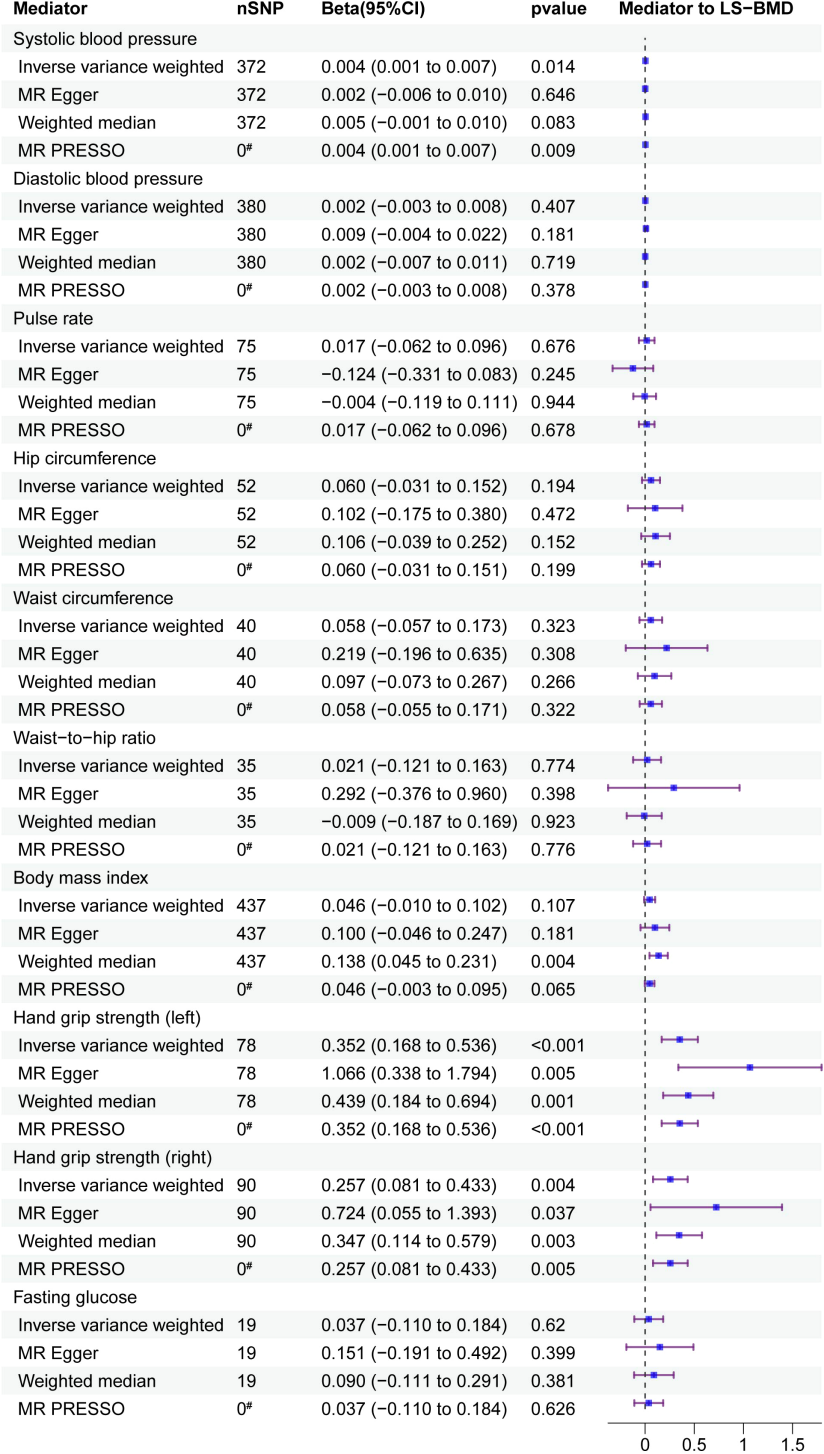
Mediator screening results. Forest plots display the relationship between HDL-C, LDL-C and TC exposure and mediator, estimated by UVMR. FAll studies in the analysis are represented in the forest plots, which contain nSNP, Beta95CI, and p-value data. ^#^ for outliers.

UVMR was employed to examine the impact of mediators on LS-BMD (**Figure 5**). The results showed a positive correlation between SBP and LS-BMD (beta: 0.004, 95% CI: 0.001 to 0.007, p = 0.014). Furthermore, L-HGS had a positive correlation with LS-BMD (beta: 0.352, 95% CI: 0.169 to 0.535, p*<*0.001), and R-HGS displayed the same trend, with a positive correlation with LS-BMD (beta: 0.257, 95% CI: 0.082 to 0.432, p = 0.004). The results obtained after conducting Cochran’s Q test indicated that there was heterogeneity in the association between R-HGS and LS-BMD, while no heterogeneity was observed in the other associations. The MR-Egger intercept test indicated the absence of horizontal pleiotropy in all associations (**Table 7**).

**Table 7:** Results of tests for heterogeneity and pleiotropy in mediators (SBP, L-HGS and R-HGS) to LS-BMD.

	Heterogeneity		Pleiotropy	
	Q	p	Intercept	p
SBP	322.723	0.966	0.001	0.569
L-HGS	97.162	0.060	-0.010	0.051
R-HGS	118.320	0.021	-0.006	0.160

### 3.6 MVMR of exposure and mediator on outcome

We analysed the direct effects of exposure on outcome after adjustment for mediators using MVMR (**Table 8**). Upon adjusting for the mediator SBP, we observed non-significant direct effects of HDL-C, LDL-C and TC on LS-BMD. Similarly, after adjusting for L-HGS, the direct effect of TC on LS-BMD was no longer significant. Additionally, after adjusting for R-HS, the direct effect of TC on LS-BMD disappeared.

**Table 8:** Results of MVMR.

Phenotype	nSNP	Beta(95% CI)	p	F-statistic
HDL-C to LS-BMD				
HDL-C, adjusted SBP	565	-0.048 (-0.164, 0.067)	0.414	28.547
SBP, adjusted HDL-C LDL-C to LS-BMD	565	0.008(0.002, 0.014)	0.011	21.043
LDL-C, adjusted SBP	542	-0.008(-0.118, 0.101)	0.885	29.595
SBP, adjusted LDL-C TC to LS-BMD	542	0.003(-0.002, 0.009)	0.198	27.026
TC, adjusted SBP	559	0.003(-0.109, 0.115)	0.955	29.621
SBP, adjusted TC	559	0.004(-0.001, 0.010)	0.121	23.952
TC, adjusted L-HGS	549	-0.081(-0.171, 0.010)	0.081	40.986
L-HGS, adjusted TC	549	-0.075(-0.318, 0.168)	0.544	4.171
TC, adjusted R-HGS	528	-0.078(-0.169, 0.013)	0.094	42.182
R-HGS, adjusted TC	528	-0.284(-0.503, -0.065)	0.011	5

### 3.7 Intermediary effect calculation

The results of the MVMR analysis shown that the direct effect of the exposure on the outcome was no longer significant once mediators have been taken into account. To evaluate the mediating effect, we utilized the coefficient product method. The calculations reveal that SBP played a mediating role in the pathway from HDL-C to LS-BMD, contributing to 3.17% of the effect; however, a suppression effect was observed in the pathways from LDL-C and TC to LS-BMD. Furthermore, L-HGS and R-HGS mediated 6.90% and 4.60% respectively, of the pathway from TC to LS-BMD (**Table 9**).

**Table 9:** Results of the indirect effect calculation.

Action	Mediator	Beta	SE	95%CI	Proportion of indirect effect
HDL-C to LS-BMD					
Total effect		-0.063	0.022	-0.107, -0.019	
Indirect effect LDL-C to LS-BMD	SBP	-0.002	0.001	-0.005, -0.001	3.17%
Total effect		-0.073	0.024	-0.121, -0.026	
Indirect effect TC to LS-BMD	SBP	0.002	0.001	0.001, 0.004	Suppression effect
Total effect		-0.087	0.023	-0.133,-0.041	
Indirect effect	SBP	0.002	0.001	0.001, 0.003	Suppression effect
Indirect effect	L-HGS	-0.006	0.002	-0.011, -0.001	6.90%
Indirect effect	R-HGS	-0.004	0.002	-0.009,-0.001	4.60%

## 4 Discussion

This large-scale study using MR analysis presented a persuasive body of new evidence supporting a causal connection between lipid and bone metabolism. The evidence revealed that augmented HDL-C, LDL-C and TC by 1 SD was linked to a decrease of 0.039 g/cm^2^, 0.045 g/cm^2^ and 0.054 g/cm^2^, respectively in LS-BMD. Conversely, no significant association was observed between lipid metabolism and FA-BMD or FN-BMD. To gain further insight, we conducted three additional studies to explore the causal relationships between lipid metabolism and bone metabolism. First, we performed a subgroup analysis of lipid metabolism data by gender and found that HDL-C and LDL-C in both male and female groups, and TC in the female group, showed a negative causal relationship with LS-BMD. However, the causal relationship between TC and LS-BMD disappeared in the male group. Furthermore, we performed reverse MR analysis and found no causal relationship between LS-BMD and lipid metabolism, supporting the unidirectional influence of lipid metabolism on bone metabolism. Finally, we conducted a meticulous investigation of potential mediators associated with common anthropometric traits along the pathway linking lipid metabolism to LS-BMD. Notably, analyses revealed that SBP account for 3.17% of the effect of HDL-C on LS-BMD, while a suppression effect occured in the pathways from LDL-C and TC to LS-BMD. In addition, L-HGS and R-HGS mediated 6.9% and 4.6% of the effect of TC on LS-BMD, respectively. In conclusion, our study demonstrated a negative association between lipid and bone metabolism and sheds light on the influential role of anthropometric characteristics on this pathway.

Currently, an increasing number of studies have demonstrated a close correlation between lipid metabolism and bone metabolism, although the conclusions remain debatable. HDL-C, acknowledged as “good” cholesterol with cardiovascular protectionJomard and Osto (23), has a disputed association with BMD. Several studies have displayed a positive correlation between HDL-C and LS-BMDXie et al. (24), Zolfaroli et al. (25), while other observational studies have established a negative association Tang et al. (26), Jiang et al. (27), Panahi et al. (28). LDL-C and TC are significant risk factors for cardiovascular disease. Previous observational studies have suggested a negative association between LDL-C and LS-BMD Alay et al. (29), Xiao et al. (30). However, there are conflicting results regarding the association between TC and BMD. Some studies have found a positive correlation or no association between TC and BMD Hernandez et al. (31), Brownbill and Ilich (32), Samelson et al. (33), Solomon et al. (34), while others have demonstrated a negative correlation between TC and LS-BMDMakovey et al. (12), Sun et al. (35), Anagnostis et al. (36), Fang et al. (37)Hu et al. (, 38). In recent years, there has been an increasing number of MR studies that have examined the causal relationship between lipid metabolism and bone metabolism. One study revealed that HDL-C is a risk factor for LS-BMD, while both HDL-C and LDL-C are risk factors for BMD Zhang et al. (39). Another MR analysis provided consistent evidence of a negative causal association between LDL-C and BMD Li et al. (40). This observation was in line with the findings of a separate MR study Zheng et al. (41). Moreover, a two-sample MR study demonstrated negative causal associations between LDL-C, TC, TG, and BMD Yang et al. (42). Furthermore, the study uncovered further insights into the causal relationship between blood lipids and fracture risk mediated by BMD. It was discovered that lower levels of HDL-C correlated negatively with LS-BMD, suggesting a potential association between reduced LS-BMD and decreased HDL-C levels. Conversely, the study showed a positive correlation between TG levels and BMD, implying that higher TG levels may be linked to increased BMD (43). The cumulative evidence suggested that lipid metabolism may have detrimental effects on bone metabolism. Our research findings revealed a negative causal association between HDL-C, LDL-C, and TC levels and LS-BMD. Some of our research conclusions are consistent with prior studies, although disparities exist regarding specific sites when compared to previous MR investigations. These variations might be ascribed to the skeletal composition of different skeletal sites (significant regional differences in cortical and trabecular bone and bone microstructure) or the influence of additional risk factors or the necessity for further exploration and advancement of the human genetic mutation database.

It is worth noting that our study uncovered the gender-specificity in the causal relationships between lipid metabolism and bone metabolism. We found that the causal relationships between HDL-C and LDL-C with LS-BMD were consistent across gender groups. However, we observed that TC had no causal relationship with LS-BMD in the male group, whereas there was a negative causal relationship in the female group. This is consistent with the findings of a cross-sectional study that reported a negative association between TC and LS-BMD in women aged 45 years and older Fang et al. (37). Another study that also performed sex-stratified subgroup analysis found a negative association between TC and LS-BMD in the female group Wang et al. (44). The results of our study confirmed previous findings.

Another significant finding of this study is the identification and quantification of human traits as mediators in the connection between lipid and bone metabolism. We examined 10 candidate mediators linked to human traits, which led us to choose SBP and HGS as the mediators. Interestingly, it was observed that the indirect impact of SBP was unfavorable in the HDL-C to LS-BMD pathway. Conversely, SBP has a suppression effect on the causal relationship between LDL-C and TC on LS-BMD. In other words, the positive regulatory influence of SBP on LS-BMD partially masks the negative effects of LDL-C and TC on LS-BMD. This phenomenon emphasises the importance of taking into account the mediating effect of SBP when analysing the negative causal relationship between LDL-C and TC with LS-BMD, as failure to do so may lead to an underestimation of the effect of LDL-C and TC on the negative causal relationship with LS-BMD (45). This finding indicated a possible interaction between the direct and indirect effects, resulting in a compensatory mechanism. Additionally, HGS displayed an adverse indirect effect in the TC to LS-BMD pathway. Consistent with previous epidemiological and MR studies, a positive association between SBP and HGS with LS-BMD was consistently observed (46–49). Additionally, HDL-C displayed a negative correlation with SBP (50), whereas LDL-C or TC demonstrated a positive correlation (51–53). It is worth noting that TC demonstrated a negative association with HGS (54).

Despite a growing number of studies investigating the link between lipid metabolism and bone metabolism, the mechanisms underlying their interaction remain uncertain. According to existing research, the association between lipid metabolism and bone metabolism could be explained by three main factors. Firstly, sex hormone levels, specifically estrogen and testosterone, are crucial in maintaining “bone homeostasis”. Secondly, inflammation, oxidative stress, and parathyroid hormone (PTH) levels are also involved in this link. Finally, insulin resistance and alterations in adipokine levels, particularly leptin and adiponectin, have been found to play a role in the relationship between lipid and bone metabolism. Estrogen helps to maintain bone density and inhibits bone resorption, while testosterone promotes bone growth and increases bone density (55–57). Several studies shown a strong negative correlation between HDL-C and sex hormones (58, 59), TC and LDL-C are negatively correlated with estrogen (60). Additionally, inflammatory responses impact bone metabolism by affecting the activation or function of osteoclasts (61, 62). HDL-C, LDL-C, and TC demonstrate positive correlations with inflammatory factors triggering osteoclast differentiation and function that disrupt the bone’s metabolic homeostasis (63, 64). Additionally, lipid oxidation products facilitate arterial calcification by activating osteoblasts in the vascular pool, while their accumulation in the periosteal endosteal space hinders bone formation (65). A study on mesenchymal stem cell differentiation has shown that HDL-C has antioxidant properties by inhibiting the accumulation of lipid oxidation products. This, in turn, affects osteogenic differentiation by removing oxygenated sterols from the surrounding tissues (66). These three factors could explain the negative correlation between lipid metabolism and bone metabolism. Nonetheless, there is inadequate direct evidence to support these hypotheses; hence, additional experiments are imperative.

This study demonstrates a sexual dimorphism in the causal relationship between TC and LS-BMD. Although there is currently no direct evidence to establish the specific mechanism, we hypothesise that estrogen levels play a crucial role in this process, as estrogen is a key steroid hormone in metabolic regulation. Its effects are mediated by estrogen receptors (ERs), the most important of which is ER*β*, encoded by the ESR2 gene. Research has suggested an association between ESR2 polymorphisms and TC levels (67), and lower ESR2 levels have also been observed in males (68). We speculate that the difference in ESR2 levels between males and females is an important factor contributing to the sexual dimorphism in the causal relationship between TC and LS-BMD.

SBP and HGS play a mediating role in lipid and bone metabolism, although the exact mechanisms of their mediation remain unclear. The mechanism behind the positive correlation between SBP and BMD is related to hormonal fluctuations. Research has shown that elevated SBP leads to an increase in several hormones in the body, such as parathyroid hormone, which plays a crucial role in bone remodelling, stimulating bone formation and increasing BMD (69, 70). It is known that HDL-C is negatively correlated with SBP, whereas LDL-C and TC are positively correlated with SBP. The process of raising or lowering SBP triggers changes in hormone levels in the body, thereby influencing BMD. This process overlaps with the mechanisms by which lipid metabolism affects bone metabolism, providing a partial explanation for the mediating role of SBP in the influence of lipid metabolism on bone metabolism pathways. HGS is an important indicator of muscle wasting. Studies have identified TC as a risk factor for muscle loss (71), with mechanisms including insulin resistance (72) and the release of inflammatory cytokines (73). In addition, research has shown that skeletal muscles secrete various myokines via autocrine, paracrine or endocrine pathways, thereby regulating the metabolic activities of bone cells in a variety of ways, ultimately affecting BMD (74). These mechanisms may represent potential pathways to explain the role of HGS in the causal relationship between TC and LS-BMD.

In our study we found a causal relationship between the levels of HDL-C, LDL-C and TC in lipid metabolism and LS-BMD. However, no such relationship was observed for FA BMD and FN BMD. The discrepancy in these findings may be due to variations in cancellous bone mass at different skeletal sites (75, 76). The strength of the lumbar spine depends primarily on cancellous bone, which not only supports the weight of the upper body but also maintains flexibility. Therefore, cancellous bone mass in the lumbar spine is relatively abundant compared to the femoral neck and forearm (77). Cancellous bone has high metabolic activity and a rapid bone turnover rate, which accelerates bone formation and remodelling, ultimately leading to increased bone density (78). Cancellous bone mass is closely linked to bone cells, osteoblasts and osteoclasts. Research has shown that inhibiting apoptosis in bone cells and osteoblasts can increase cancellous bone mass (79). Lipid metabolism may also affect cancellous bone mass. Studies have suggested that peroxisome proliferator-activated receptor *γ* (PPAR*γ*), which is activated by lipid metabolism products, as well as lipid oxidation products, can inhibit osteoblast differentiation, resulting in reduced cancellous bone mass (28). In addition, low-density lipoprotein receptor (LDLR) deficiency has been associated with impaired LDL-C clearance, resulting in elevated blood LDL levels. This deficiency also activates osteoclast activity, leading to disruption of trabecular bone microstructure and reduced cancellous bone mass (80).

DXA is considered the benchmark for evaluating bone density and identifying the initial signs of bone metabolism disturbances. Nevertheless, despite its efficacy, numerous nations have not yet adopted DXA as a standard element of medical screening procedures. Currently, basic screening techniques to recognise bone metabolism disorders mostly depend on population-based strategies, such as age, susceptibility fractures, perimenopausal status, or opportunistic testing. Nonetheless, these techniques possess restrictions regarding accuracy and cost-effectiveness. Conversely, traditional health screenings emphasizing lipid profiling and anthropometric measurements offer a crucial means for timely disease identification. Our research provides fresh observations on the prevention and diagnosis of bone metabolic ailments by studying the causal link between lipid profiling, anthropometric measurements, and LS-BMD within customary health screenings.

This study elucidated the causal relationships between blood lipids and BMD in different anatomical sites and identifies causal mediators in the pathways between lipid metabolism and bone metabolism through MR research. The study has several strengths. Firstly, we utilized the largest available lipid and BMD GWAS data, guaranteeing minimal overlap between exposures, mediators, and outcomes and thus maintaining a low type 1 error rate. Secondly, MR-PRESSO and Steiger-filtering tests were conducted to consider potential pleiotropic effects. The identification of outliers, if any, did not undermine the causal effects detected in the original IVW analysis. Thirdly, several MR sensitivity analyses were employed in this study to support the reliability of IVW estimates, each accounting for different assumptions regarding genetic pleiotropy (81). Fourthly, we implemented stringent criteria for mediator selection to ensure the credibility and plausibility of the constructed models elucidating mediation effects. However, this study also has certain limitations. Firstly, although we focused on common and clinically relevant human traits as potential mediators driving clinical practice, we were unable to fully explain the mediating effects between lipid metabolism and bone metabolism. For instance, specific mediators such as menopausal status and age at menarche in females remain unaccounted for (82, 83). Secondly, the persisting heterogeneity of SNPs could introduce bias and compromise the reliability of MR results. Thirdly, the majority of the GWAS used in this study primarily included European populations from high-income countries. Therefore, further investigations are required to extend the generalizability of our findings to other ethnic groups, as well as low- and middle-income countries.

In conclusion, this study using MR has provided insight into the negative causal relationship between lipid and bone metabolism. It also indicated the causal mediators through which blood lipids affect bone density. The study offeres causal evidence for the pathogenesis of bone metabolism disorders, which facilitates early prevention and diagnosis of such disorders.

## Data availability statement

The original contributions presented in the study are included in the article/**Supplementary Material**. Further inquiries can be directed to the corresponding author.

## Author contributions

JK: Conceptualization, Data curation, Formal Analysis, Investigation, Methodology, Project administration, Resources, Software, Validation, Visualization, Writing – original draft, Supervision. SZ: Conceptualization, Investigation, Methodology, Resources, Software, Validation, Visualization, Writing – review & editing. XW: Methodology, Supervision, Validation, Writing – review & editing. CW: Data curation, Methodology, Supervision, Validation, Writing – review & editing. ZJ: Conceptualization, Data curation, Formal Analysis, Methodology, Project administration, Resources, Supervision, Validation, Writing – review & editing. SW: Formal Analysis, Funding acquisition, Methodology, Project administration, Resources, Supervision, Validation, Writing – review & editing.

## Supplementary material

Supplementary Figure 1 Scatterplot, funnel plot and leave-one-out analysis of relationship between HDL-C and LSBMD.

Supplementary Figure 2 Scatterplot, funnel plot and leave-one-out analysis of relationship between LDL-C and LS-BMD.

Supplementary Figure 3 Scatterplot, funnel plot and leave-one-out analysis of relationship between TC and LS-BMD.

Supplementary Figure 4 Scatterplot, funnel plot and leave-one-out analysis of relationship between HDL-C and SBP.

Supplementary Figure 5 Scatterplot, funnel plot and leave-one-out analysis of relationship between LDL-C and SBP.

Supplementary Figure 6 Scatterplot, funnel plot and leave-one-out analysis of relationship between TC and SBP.

Supplementary Figure 7 Scatterplot, funnel plot and leave-one-out analysis of relationship between TC and L-HGS.

Supplementary Figure 8 Scatterplot, funnel plot and leave-one-out analysis of relationship between TC and R-HGS.

Supplementary Figure 9 Scatterplot, funnel plot and leave-one-out analysis of relationship between SBP and LS-BMD.

Supplementary Figure 10 Scatterplot, funnel plot and leave-one-out analysis of relationship between L-HGS and LS-BMD.

Supplementary Figure 11 Scatterplot, funnel plot and leave-one-out analysis of relationship between R-HGS and LS-BMD.

Supplementary Table 1 Instrumental variables for HDL-C to LS-BMD.

Supplementary Table 2 Instrumental variables for LDL-C to LS-BMD.

Supplementary Table 3 Instrumental variables for TC to LS-BMD.

Supplementary Table 4 MVMR of HDL-C and SBP to LS-BMD with instrumental variables.

Supplementary Table 5 MVMR of LDL-C and SBP to LS-BMD with instrumental variables.

Supplementary Table 6 MVMR of TC and SBP to LS-BMD with instrumental variables.

Supplementary Table 7 MVMR of TC and L-HGS to LS-BMD with instrumental variables.

Supplementary Table 8 MVMR of TC and R-HGS to LS-BMD with instrumental variables.
