# Supplementary material for: The association of lipid metabolism with bone metabolism and the role of human traits: a Mendelian randomization study
Source: Front Endocrinol (Lausanne). 2023 Dec 6;14:1271942. doi: 10.3389/fendo.2023.1271942 (PMC10731031; doi:10.3389/fendo.2023.1271942)
Supplement: Supplementary file 1 [file DataSheet_1.docx]

**STROBE-MR checklist of recommended items to address in reports of Mendelian randomization studies**^1^ ^2^

| **Item No.** | **Section** | **Checklist item** | **Page No.** | **Relevant text from manuscript** |
| --- | --- | --- | --- | --- |
| 1 | **TITLE and ABSTRACT** | Indicate Mendelian randomization (MR) as the study’s design in the title and/or the abstract if that is a main purpose of the study | 1 | The association of lipid metabolism with bone metabolism and the role of human traits: A Mendelian Randomization Study |
|  | **INTRODUCTION** |  |  |  |
| 2 | **Background** | Explain the scientific background and rationale for the reported study. What is the exposure? Is a potential causal relationship between exposure and outcome plausible? Justify why MR is a helpful method to address the study question | 4 | Lipid metabolism is the process of synthesis, degradation, and transport for lipids in various tissues of the body and plays a crucial role as a regulatory messenger in systemic metabolism. It is closely related to bone metabolism, and lipid metabolism disorders can directly affect bone formation and absorption, thereby affecting bone mineral density (BMD) and strength. Despite the increasing number of studies on the relationship between lipid metabolism and bone metabolism, the results are still inconsistent. Therefore, the use of MR to investigate whether there is a correlation between the above lipid metabolism and bone metabolism. The ultimate goal is to prevent or delay the progression of the disease. |
| 3 | **Objectives** | State specific objectives clearly, including pre-specified causal hypotheses (if any). State that MR is a method that, under specific assumptions, intends to estimate causal effects | 4 | This study investigates the causal associations between lipids and bone metabolism using a two-sample Mendelian randomization approach. Additionally, it explores the role of human traits in the pathways linking lipid and bone metabolism, making a valuable contribution to the field of metabolism. |
|  | **METHODS** |  |  |  |
| 4 | **Study design and data sources** | Present key elements of the study design early in the article. Consider including a table listing sources of data for all phases of the study. For each data source contributing to the analysis, describe the following: | 5 | In this study, genetic variables associated with BMD were derived from a comprehensive summary of the largest publicly available GWAS meta-analysis conducted by the Osteoporosis Consortium, which focused on individuals of European ethnicity (GEFOS, http://www.gefos.org/?q=content/data-release-2015). In order to identify genetic variables associated with lipid mass spectrometry, we utilized data from the Global Federation of Lipid Genetics Consortium (GLGC, https://csg.sph.umich.edu/willer/public/glgc-lipids2021/) comprising a sample size of 1,320,016 individuals. To address any potential biases arising from sample overlap, we selected data from multiple consortia involved in genome-wide association studies (GWAS) as the source of these mediators (See Table 1 for specific information). |
|  | a) | Setting: Describe the study design and the underlying population, if possible. Describe the setting, locations, and relevant dates, including periods of recruitment, exposure, follow-up, and data collection, when available. | 6-8 | Exposure data for this study were obtained from the Global Lipid Alliance, outcome data from the Osteoporosis Consortium, and mediator data from the MRCIEU database. The study subjects were all European. |
|  | b) | Participants: Give the eligibility criteria, and the sources and methods of selection of participants. Report the sample size, and whether any power or sample size calculations were carried out prior to the main analysis | 6,7 | Studies reported Osteoporosis Alliance sample sizes of FA-BMD (n=10,805), LS-BMD (n=44,731), and FN-BMD (n=49,988)，Adjustments were made for gender, age, and BMI, and the data underwent weighting and standardization to achieve a mean of 0 and a standard deviation of 1.  The lipid sample size is 1,320,016. Adjusted for age, age2, sex, principal components, and any necessary study-specific covariates. |
|  | c) | Describe measurement, quality control and selection of genetic variants | 8 | we established a genome-wide significance threshold of p<5×108 for screening single nucleotide polymorphisms (SNPs) associated with exposure factors. One potential problem arising from linkage disequilibrium (LD) is the impact on SNPs used to capture causal changes, as they may be influenced by other confounding factors, thereby violating the second or third IVs hypothesis. To mitigate the adverse effects of LD, we used a clustering approach (R2<0.001, kb=10000). To assess the strength of the selected SNPs and to minimize weak instrumental bias, we calculated the F-statistic. SNPs were considered as non-weak instruments if their F-statistic was >10 and their minor allele frequency (MAF) was >0.01. |
|  | d) | For each exposure, outcome, and other relevant variables, describe methods of assessment and diagnostic criteria for diseases | 7, | BMD was measured using Dual-energy X-ray absorptiometry (DXA)..Triglyceride levels were natural logarithm transformed before generating residuals, which were then inverse normalized. The pre-medication levels of individuals taking cholesterol-lowering drugs were approximated by dividing LDL-C values by 0.7 and TC values by 0.8. |
|  | e) | Provide details of ethics committee approval and participant informed consent, if relevant | Yes | The study was ethically cleared and the ethical approval is shown in the supplementary file |
| 5 | **Assumptions** | Explicitly state the three core IV assumptions for the main analysis (relevance, independence and exclusion restriction) as well assumptions for any additional or sensitivity analysis | 8,9 | The three assumptions of IVs in MR analysis: (1) Strong correlation with the exposure factor; (2) Independence from confounding factors; (3) The IVs affect the outcome through the exposure factor rather than through other means. In addition, three methods of sensitivity analyses and horizontal polytropy tests are described 4 methods for sensitivity analyses and horizontal multiplicity testing: IVM, weighted median method, MR-Egger and MR-PRESSO, and report the use of the F-statistic to calculate statistical validity and Q-statistic to detect heterogeneity. and Q-statistics to detect heterogeneity |
| 6 | **Statistical methods: main analysis** | Describe statistical methods and statistics used |  |  |
|  | a) | Describe how quantitative variables were handled in the analyses (i.e., scale, units, model) | 9 | Reported analyses using random-effects models when there were >3 instrumental variables for genetic variants, otherwise fixed-effects models were used; statistical effect sizes or units of measure for exposures, outcomes were not transformed and therefore not reported |
|  | b) | Describe how genetic variants were handled in the analyses and, if applicable, how their weights were selected | 8 | To assess the strength of the selected SNPs and to minimize weak instrumental bias, we calculated the F-statistic. SNPs were considered as non-weak instruments if their F-statistic was >10 and their minor allele frequency (MAF) was >0.01. This stringent criterion ensured that the results of MR analysis were not unduly influenced by weak instrument bias.  To address potential bias due to reverse causality (in which SNPs are more strongly correlated with outcomes than with exposures), we used the Steiger Flering method to filter SNPs. In addition, we employed the MR-PRESSO (Mendelian randomized multivariate residual sums and outliers) test to identify multi-instrument pooled MR tests at the level of SNPs potential outliers with pleiotropic effects. |
|  | c) | Describe the MR estimator (e.g. two-stage least squares, Wald ratio) and related statistics. Detail the included covariates and, in case of two-sample MR, whether the same covariate set was used for adjustment in the two samples | 7 | Studies reported Osteoporosis Alliance sample sizes of FA-BMD (n=10,805), LS-BMD (n=44,731), and FN-BMD (n=49,988)，Adjustments were made for gender, age, and BMI, and the data underwent weighting and standardization to achieve a mean of 0 and a standard deviation of 1.  The lipid sample size is 1,320,016. Adjusted for age, age2, sex, principal components, and any necessary study-specific covariates. |
|  | d) | Explain how missing data were addressed | No | Not mentioned |
|  | e) | If applicable, indicate how multiple testing was addressed | 9 | In the UVMR analyses, Bonferroni correction was used for multiple comparisons with a significance threshold of 0.05/4 = 0.0125. This means that exposure on outcome associations with a p-value < 0.0125 were considered to have a causal effect. |
| 7 | **Assessment of assumptions** | Describe any methods or prior knowledge used to assess the assumptions or justify their validity | 9 | Although a growing number of studies have shown that disorders of lipid metabolism affect bone metabolic homeostasis, the conclusions have been inconsistent. Using F-statistics to estimate statistical validity |
| 8 | **Sensitivity analyses and additional analyses** | Describe any sensitivity analyses or additional analyses performed (e.g. comparison of effect estimates from different approaches, independent replication, bias analytic techniques, validation of instruments, simulations) | 9,10 | Three methods for detecting sensitivity and horizontal multiple validity: weighted median, MR-Egger and MRPRESSO, using F-statistics to assess statistical validity and Q-statistics to measure to detect heterogeneity |
| 9 | **Software and pre-registration** |  |  |  |
|  | a) | Name statistical software and package(s), including version and settings used | 10 | All MR analyses were performed with R software (version 4.0.2; R Foundation for Statistical Computing, Vienna, Austria) and the R packages “TwoSampleMR”, “MendelianRandomization”, “MRPRESSO” and “MVMR”. |
|  | b) | State whether the study protocol and details were pre-registered (as well as when and where) | No | This study uses public databases, is not registered and does not have a report plan |
|  | **RESULTS** |  |  |  |
| 10 | **Descriptive data** |  |  |  |
|  | a) | Report the numbers of individuals at each stage of included studies and reasons for exclusion. Consider use of a flow diagram | No | The methodology section provides the sample population number, no information on the population is provided in the results. Information on excluded populations and reasons for exclusion was not provided, and no flowchart was used. information about the excluded populations and reasons for exclusion, and no flowchart was used |
|  | b) | Report summary statistics for phenotypic exposure(s), outcome(s), and other relevant variables (e.g. means, SDs, proportions) | Yes | Information is provided in Table 1, which presents detailed information on the characteristics of the study population, exposures and mediating factors. |
|  | c) | If the data sources include meta-analyses of previous studies, provide the assessments of heterogeneity across these studies | No | The data for this study were obtained from public databases, and I^2^ values and 95% CIs were not reported to assess heterogeneity between the original studies. |
|  | d) | For two-sample MR:  i.  Provide justification of the similarity of the genetic variant-exposure associations between the exposure and outcome samples  ii.  Provide information on the number of individuals who overlap between the exposure and outcome studies | No | The two sample MRs, listed in Table 1, all sample populations are from Europe and therefore ethnically very heterogeneous, adjusting for age, gender, etc. Information on overlapping populations was not reported and there were no overlapping subjects, so they are not reported. |
| 11 | **Main results** |  |  |  |
|  | a) | Report the associations between genetic variant and exposure, and between genetic variant and outcome, preferably on an interpretable scale | 12 | We established a genome-wide significance threshold of p<5×108 for screening single nucleotide polymorphisms (SNPs) associated with exposure factors. To address potential bias due to reverse causality (in which SNPs are more strongly correlated with outcomes than with exposures), we used the Steiger Flering method to filter SNPs. |
|  | b) | Report MR estimates of the relationship between exposure and outcome, and the measures of uncertainty from the MR analysis, on an interpretable scale, such as odds ratio or relative risk per SD difference |  | As shown of the results (Figure 2), the beta values for HDL-C to LS-BMD were -0.063 (95% CI: -0.107 to -0.019; p=0.006), for LDL-C to LS-BMD were -0.073 (95% CI: -0.121 to -0.026; p=0.002) and for TC to LS-BMD were -0.087 (95% CI: -0.133 to -0.041; p<0.001). |
|  | c) | If relevant, consider translating estimates of relative risk into absolute risk for a meaningful time period | 14 | The results show that a 1SD increase in HDL-C, LDL-C and TC was associated with a decrease in LS-BMD of 0.039 g/cm2, 0.045 g/cm2 and 0.054 g/cm2, respectively. |
|  | d) | Consider plots to visualize results (e.g. forest plot, scatterplot of associations between genetic variants and outcome versus between genetic variants and exposure) | Yes | Figure 2: Forest map of exposure to outcome.  Figure 3: Forest map of exposure to mediator.  Figure 4: Forest map of mediator to outcome. |
| 12 | **Assessment of assumptions** |  |  |  |
|  | a) | Report the assessment of the validity of the assumptions | Yes | The heterogeneity of the statistical model was tested with the Q-statistic in Table 2, Table 3 and Table 4 to assess the its stability. The Annex 1 reports the statistical validity of the IVs in each association, with results expressed as F-statistics. |
|  | b) | Report any additional statistics (e.g., assessments of heterogeneity across genetic variants, such as *I^2^*, Q statistic or E-value) | Yes | The heterogeneity of the statistical model was tested with the Q-statistic in Table 2, Table 3 and Table 4 to assess the its stability |
| 13 | **Sensitivity analyses and additional analyses** |  |  |  |
|  | a) | Report any sensitivity analyses to assess the robustness of the main results to violations of the assumptions | 12 | MR analysis results obtained using four analytical methods, including IVW, weighted median method, MREgger and MR-PRESSO, Specific results can be found at Figure 2, Figure 3, Figure 4 |
|  | b) | Report results from other sensitivity analyses or additional analyses | 33-35 | The heterogeneity of the statistical model was tested with the Q-statistic in Table 2, Table 3 and Table 4 to assess the its stability. |
|  | c) | Report any assessment of direction of causal relationship (e.g., bidirectional MR) | 12 | To address potential bias due to reverse causality (in which SNPs are more strongly correlated with outcomes than with exposures), we used the Steiger Flering method to filter SNPs. |
|  | d) | When relevant, report and compare with estimates from non-MR analyses | No | Non-MR analyses were not performed in this study |
|  | e) | Consider additional plots to visualize results (e.g., leave-one-out analyses) | 30 | See figure 2 figure 3 figure 4. |
|  | **DISCUSSION** |  |  |  |
| 14 | **Key results** | Summarize key results with reference to study objectives | 15 | This large-scale MR study provides compelling new evidence supporting a causal relationship between lipid and bone metabolism. The results show that a 1SD increase in HDL-C, LDL-C and TC was associated with a decrease in LS-BMD of 0.039 g/cm2, 0.045 g/cm2 and 0.054 g/cm2, respectively. Conversely, no significant association was observed between lipid metabolism and FA-BMD or FN-BMD. To gain further insight, we conducted an in-depth investigation of the potential mediators associated with common anthropometric traits along the pathway linking lipid metabolism to LS-BMD. Notably, our analyses revealed that SBP accounted for 3.17%, 2.74% and 2.30% of the effects of HDL-C, LDL-C and TC on LS-BMD, respectively. In addition, L-HGS and R-HGS mediated 6.9% and 4.6% of the effect of TC on LS-BMD, respectively. In conclusion, our study demonstrates a negative association between lipid metabolism and bone metabolism and sheds light on the influential role of anthropometric characteristics along this pathway. |
| 15 | **Limitations** | Discuss limitations of the study, taking into account the validity of the IV assumptions, other sources of potential bias, and imprecision. Discuss both direction and magnitude of any potential bias and any efforts to address them | 17 | However, this study also has certain limitations. Firstly, although we focused on common and clinically relevant human traits as potential mediators driving clinical practice, we were unable to fully explain the mediating effects between lipid metabolism and bone metabolism. For instance, specific mediators such as menopausal status and age at menarche in females remain unaccounted for. Secondly, the persisting heterogeneity of SNPs may introduce bias and compromise the robustness of MR results. Thirdly, the majority of the GWAS used in this analysis predominantly involved European populations from high-income countries. Therefore, further investigations are required to extend the generalizability of our findings to other ethnic groups, as well as low- and middle-income countries. |
| 16 | **Interpretation** |  |  |  |
|  | a) | Meaning: Give a cautious overall interpretation of results in the context of their limitations and in comparison with other studies | 19 | Some studies have shown a positive correlation between HDL-C and LS-BMD, while other observational studies have found a negative association. LDL-C and TC are important cardiovascular risk factors, and previous observational studies have suggested a negative association between LDL-C and LS-BMD. However, the relationship between TC and BMD has yielded divergent results. Some studies have found a positive correlation or no association between TC and BMD, while others have demonstrated a negative correlation between TC and LS-BMD. In recent years, there has been an increasing number of MR studies investigating the causal relationship between lipid metabolism and bone metabolism. One MVMR study revealed that HDL-C is a risk factor for LS-BMD, while both HDL-C and LDL-C are risk factors for BMD. Another MR analysis further explored the causal relationship between LDL-C and BMD, providing consistent evidence of a negative causal association between LDL-C and BMD. This observation is in line with the findings of a separate MR study. Moreover, a two-sample MR study demonstrated negative causal associations between LDL-C, TC, TG, and BMD. Furthermore, an investigation into the causal relationship between blood lipids and fracture risk mediated by BMD revealed additional insights. Specifically, there was a negative correlation between HDL-C and LS-BMD, indicating that lower HDL-C levels may be associated with reduced LS-BMD. Conversely, the study showed a positive correlation between TG levels and BMD, implying that higher TG levels may be linked to increased BMD. The cumulative evidence suggests that lipid metabolism may have detrimental effects on bone metabolism. Our research findings reveal a negative causal association between HDL-C, LDL-C, and TC levels and LS-BMD. Some of our research conclusions are consistent with prior studies, although disparities exist regarding specific sites when compared to previous MR investigations. These variations might be ascribed to the skeletal composition of different skeletal sites (significant regional differences in cortical and trabecular bone and bone microstructure) or the influence of additional risk factors or the necessity for further exploration and advancement of the human genetic mutation database. |
|  | b) | Mechanism: Discuss underlying biological mechanisms that could drive a potential causal relationship between the investigated exposure and the outcome, and whether the gene-environment equivalence assumption is reasonable. Use causal language carefully, clarifying that IV estimates may provide causal effects only under certain assumptions | 17,18 | Despite an increasing number of studies exploring the relationship between lipid metabolism and bone metabolism, the mechanisms underlying their interaction remain uncertain. Based on existing research, the relationship between lipid metabolism and bone metabolism can be elucidated from the following three aspects. Firstly, sex hormone levels play a crucial role in maintaining "bone homeostasis," with estrogen and testosterone being particularly essential. Estrogen helps maintain bone density and inhibits bone resorption, while testosterone promotes bone growth and increases bone density. Some studies have shown a strong negative correlation between HDL-C and sex hormones, TC and LDL-C is negatively correlated with estrogen. Secondly, inflammatory responses have been shown to impact bone metabolism by affecting the activation or function of osteoclasts. HDL-C, LDL-C and TC are positively correlated with inflammatory factors that activate osteoclast differentiation and function and disrupt the metabolic homeostasis of bone. Thirdly, lipid oxidation products promote arterial calcification by activating osteoblasts in the vascular pool, while their accumulation in the periosteal endosteal space inhibits bone formationA study on mesenchymal stem cell differentiation demonstrated that HDL-C exhibits antioxidant properties by inhibiting the accumulation of lipid oxidation products, thereby influencing osteogenic differentiation by removing oxygenated sterols from surrounding tissues. |
|  | c) | Clinical relevance: Discuss whether the results have clinical or public policy relevance, and to what extent they inform effect sizes of possible interventions | 19 | The persisting heterogeneity of SNPs may introduce bias and compromise the robustness of MR results, but conclusions can still be drawn |
| 17 | **Generalizability** | Discuss the generalizability of the study results (a) to other populations, (b) across other exposure periods/timings, and (c) across other levels of exposure | 19 | The majority of the GWAS used in this analysis predominantly involved European populations from high-income countries., it limits the generalisability of the MR results to other populations |
|  | **OTHER INFORMATION** |  |  |  |
| 18 | **Funding** | Describe sources of funding and the role of funders in the present study and, if applicable, sources of funding for the databases and original study or studies on which the present study is based | 21 | This research received financial support from National Famous Old Chinese Medicine Experts' Inheritance Studio Construction Project of the State Administration of Traditional Chinese Medicine (State Administration of Traditional Chinese Medicine Human Education Letter [2022] No. 75. |
| 19 | **Data and data sharing** | Provide the data used to perform all analyses or report where and how the data can be accessed, and reference these sources in the article. Provide the statistical code needed to reproduce the results in the article, or report whether the code is publicly accessible and if so, where | 22 | The datasets generated during and/or analyzed during the current study are not publicly available but are available from the corresponding author on reasonable request. |
| 20 | **Conflicts of Interest** | All authors should declare all potential conflicts of interest | 21 | The authors declare no conflicts of interest related to this research. We have no financial, professional, or personal relationships that could potentially bias or influence the outcomes or interpretation of the study. |

This checklist is copyrighted by the Equator Network under the Creative Commons Attribution 3.0 Unported (CC BY 3.0) license.

1. Skrivankova VW, Richmond RC, Woolf BAR, Yarmolinsky J, Davies NM, Swanson SA, et al. Strengthening the Reporting of Observational Studies in Epidemiology using Mendelian Randomization (STROBE-MR) Statement. JAMA. 2021;under review.

2. Skrivankova VW, Richmond RC, Woolf BAR, Davies NM, Swanson SA, VanderWeele TJ, et al. Strengthening the Reporting of Observational Studies in Epidemiology using Mendelian Randomisation (STROBE-MR): Explanation and Elaboration. BMJ. 2021;375:n2233.
