# Supplementary material for: The association of lipid metabolism with bone metabolism and the role of human traits: a Mendelian randomization study
Source: Front Endocrinol (Lausanne). 2023 Dec 6;14:1271942. doi: 10.3389/fendo.2023.1271942 (PMC10731031; doi:10.3389/fendo.2023.1271942)
Supplement: Supplementary Figure 1 — Scatterplot, funnel plot and leave-one-out analysis of relationship between HDL-C and LSBMD. [file Table_1.docx]

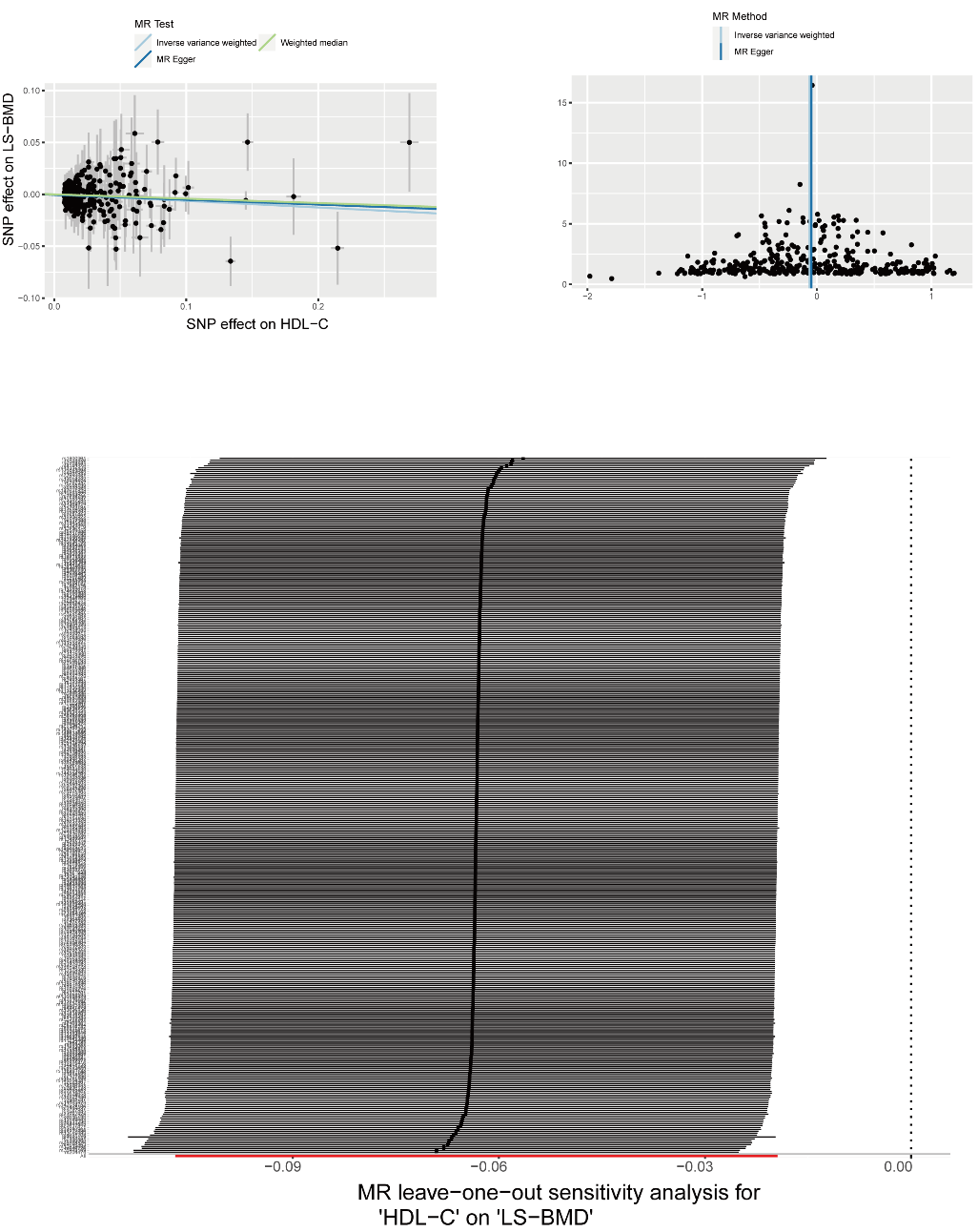


Figure S1 Scatterplot, funnel plot and leave-one-out analysis of relationship between HDL-C and LS-BMD.


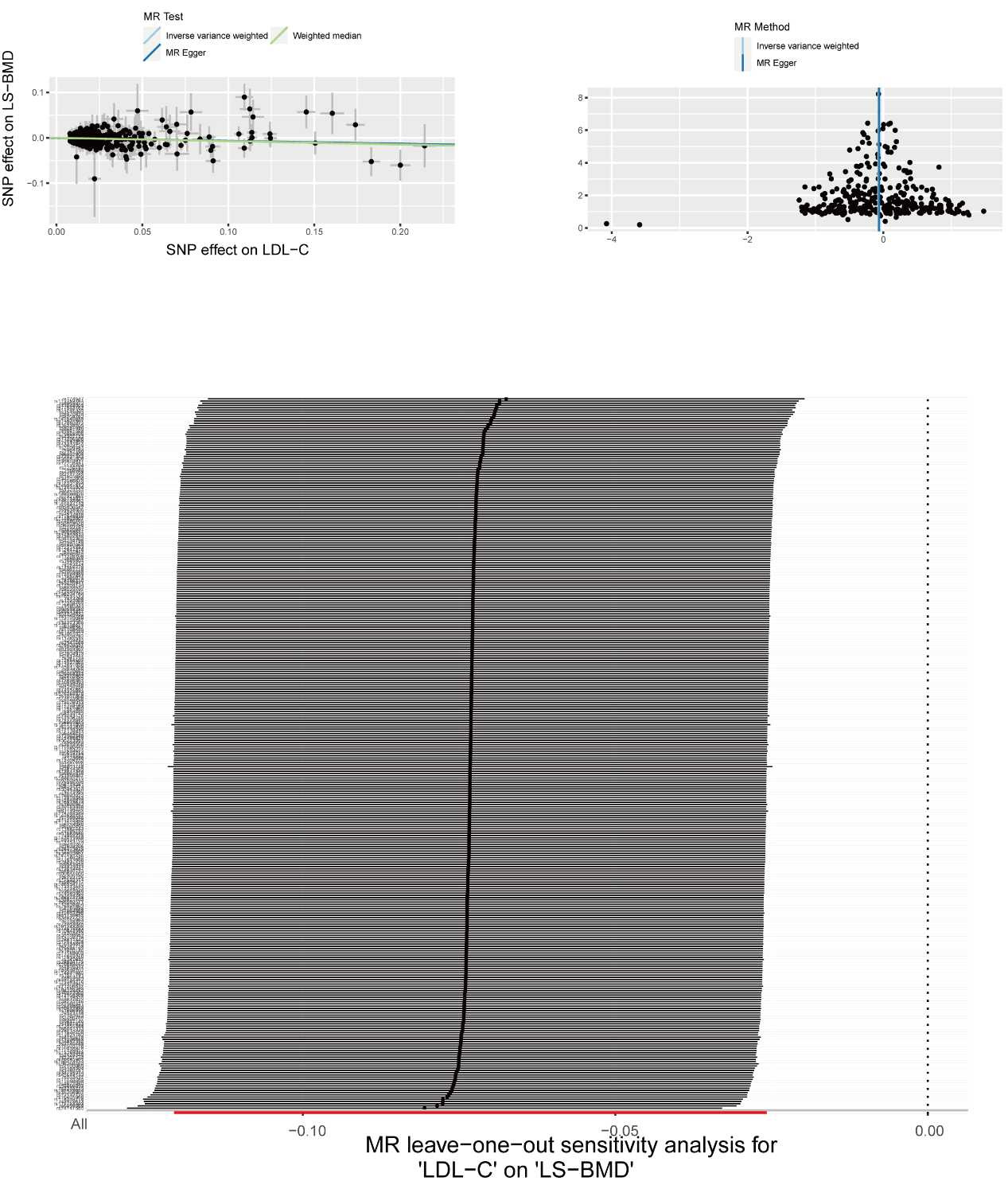


Figure S2 Scatterplot, funnel plot and leave-one-out analysis of relationship between LDL-C and LS-BMD.


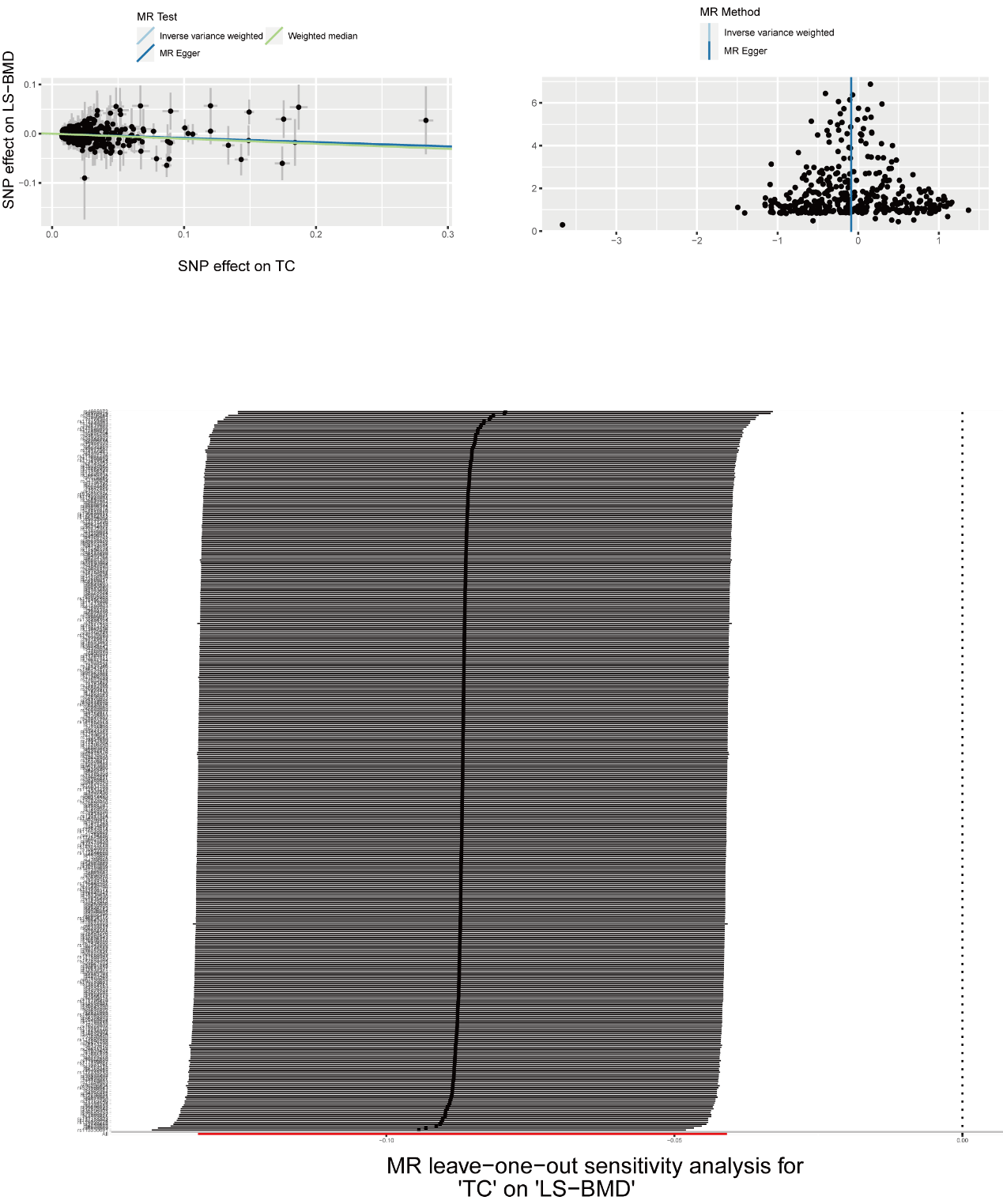


Figure S3 Scatterplot, funnel plot and leave-one-out analysis of relationship between TC and LS-BMD.


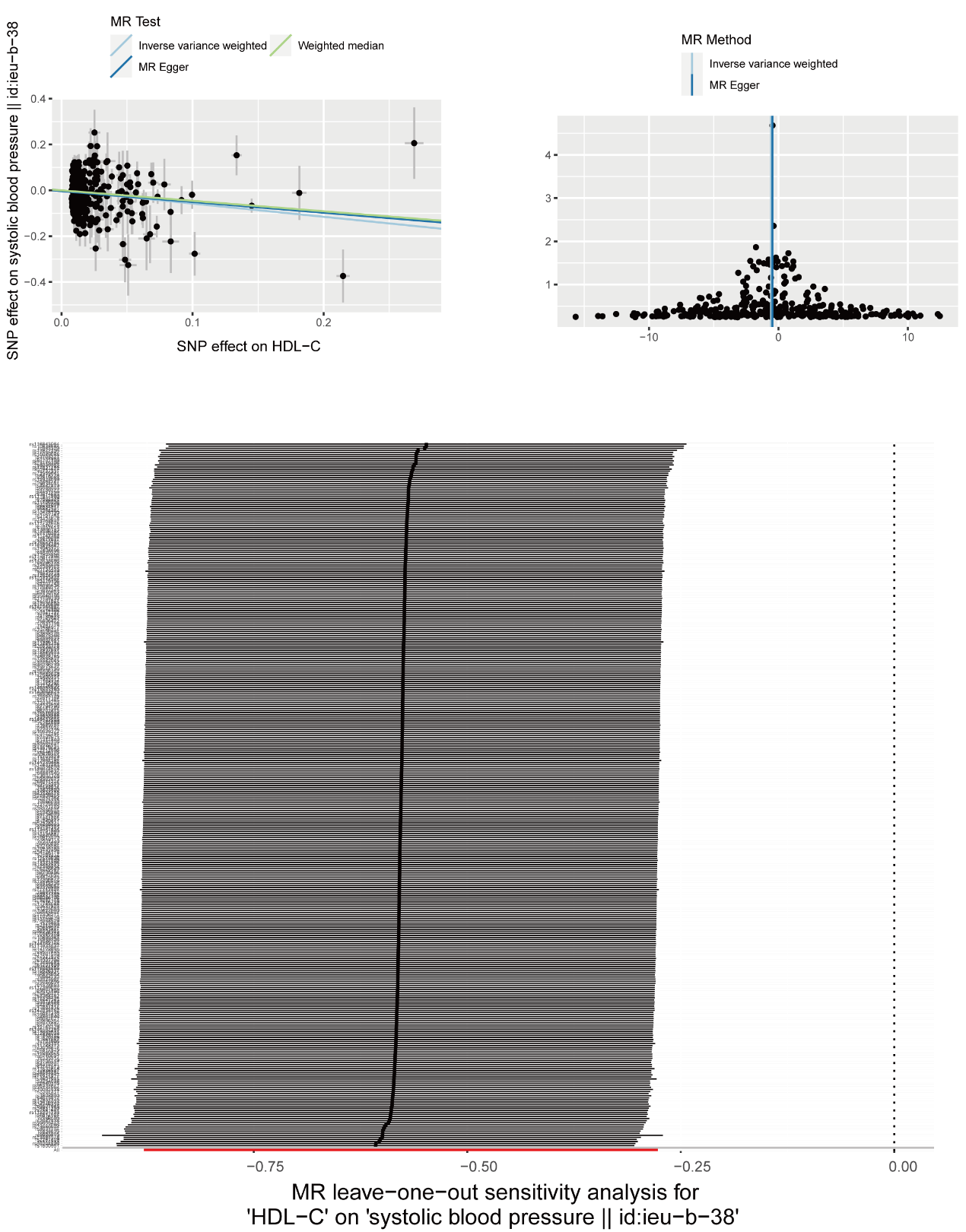


Figure S4 Scatterplot, funnel plot and leave-one-out analysis of relationship between HDL-C and SBP.


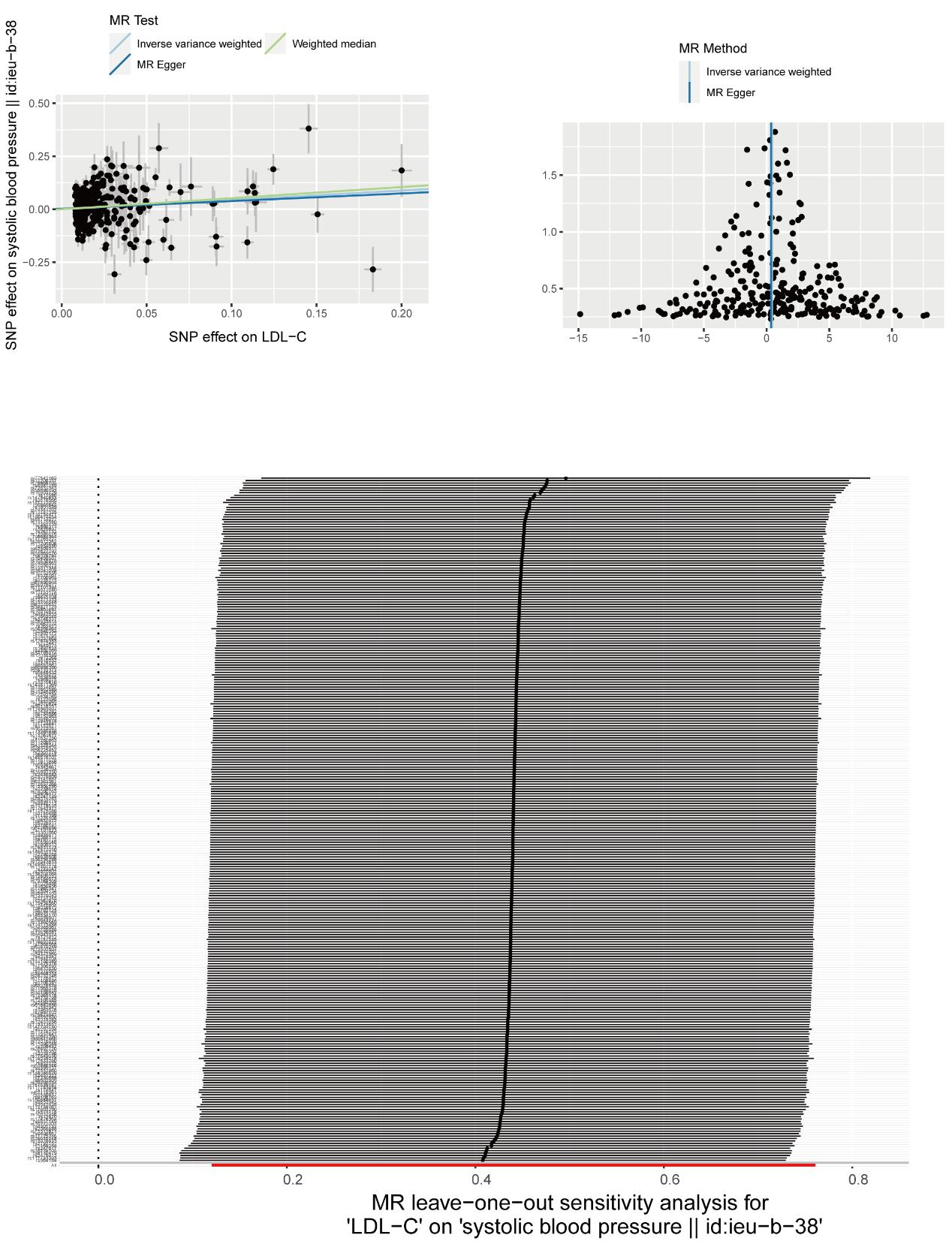


Figure S5 Scatterplot, funnel plot and leave-one-out analysis of relationship between LDL-C and SBP.


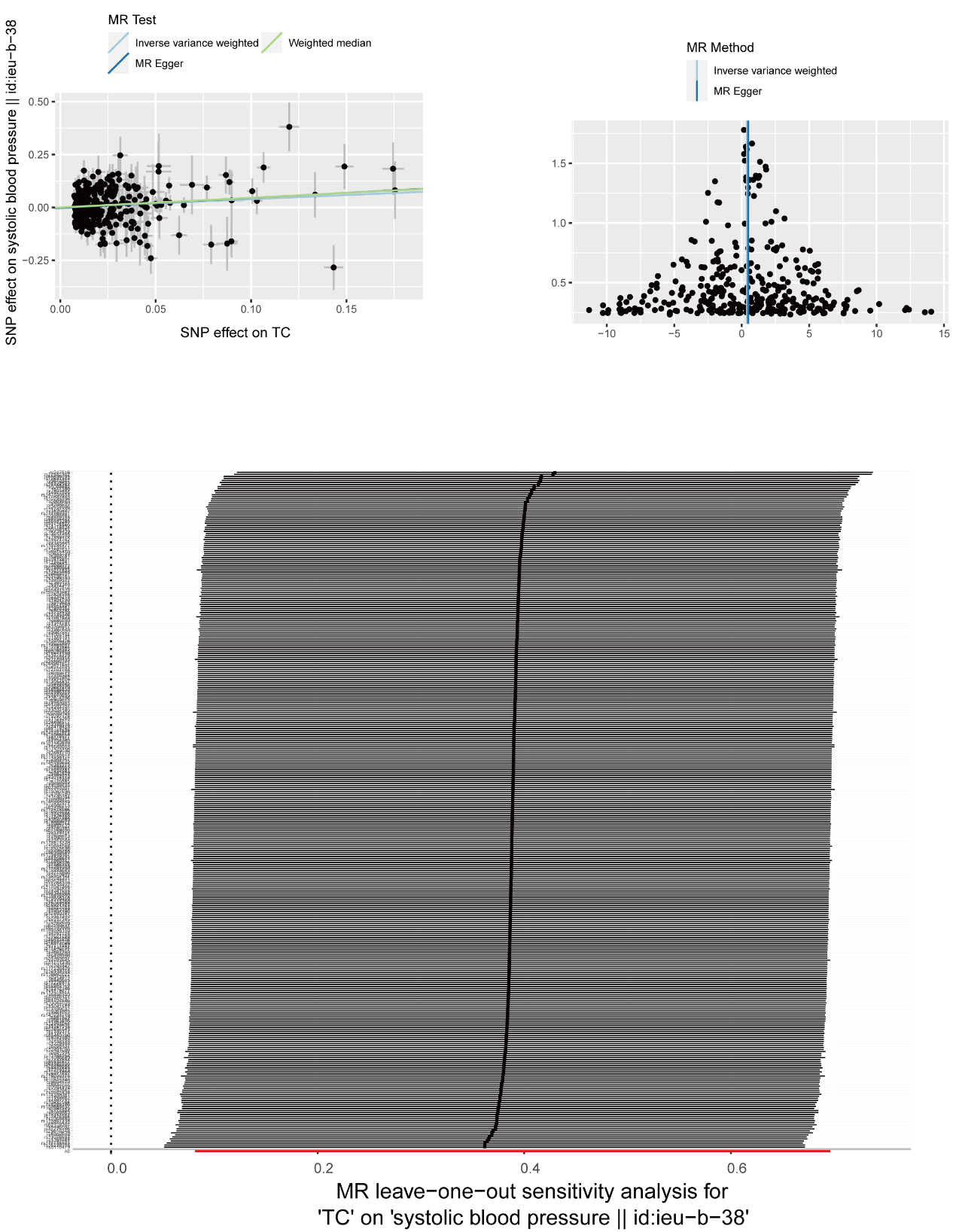


Figure S6 Scatterplot, funnel plot and leave-one-out analysis of relationship between TC and SBP.


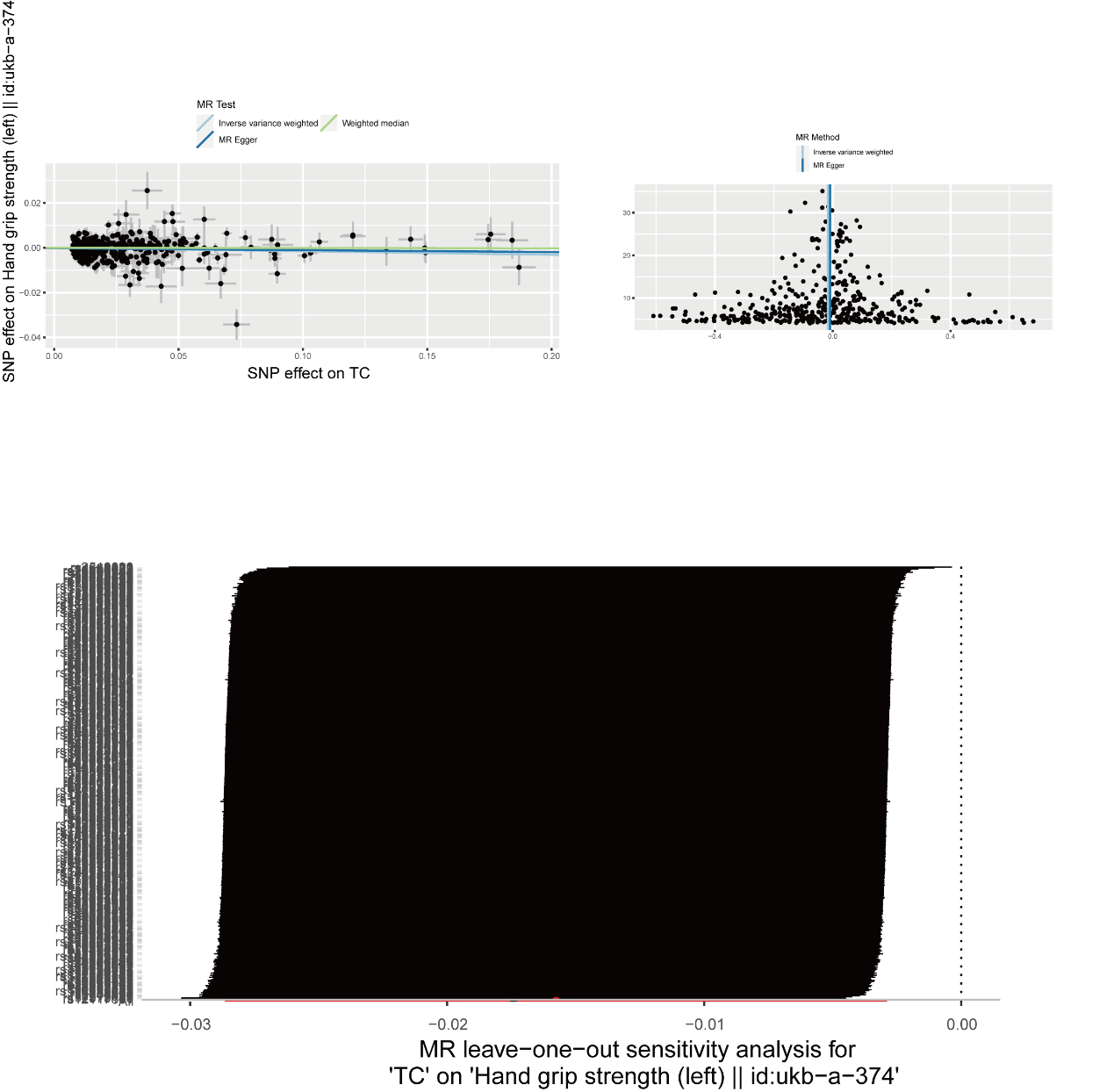


Figure S7 Scatterplot, funnel plot and leave-one-out analysis of relationship between TC and L-HGS.


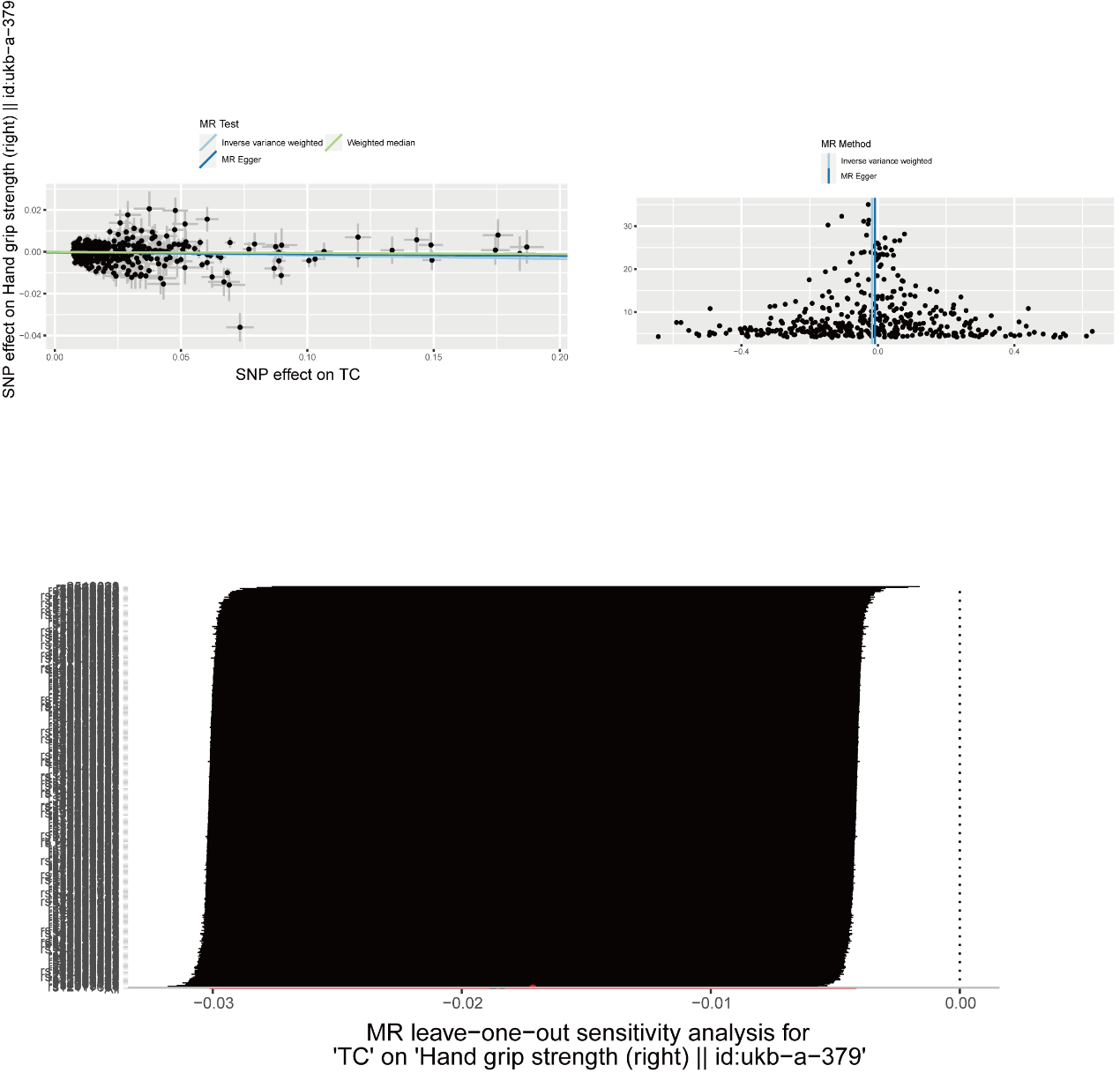


Figure S8 Scatterplot, funnel plot and leave-one-out analysis of relationship between TC and R-HGS.


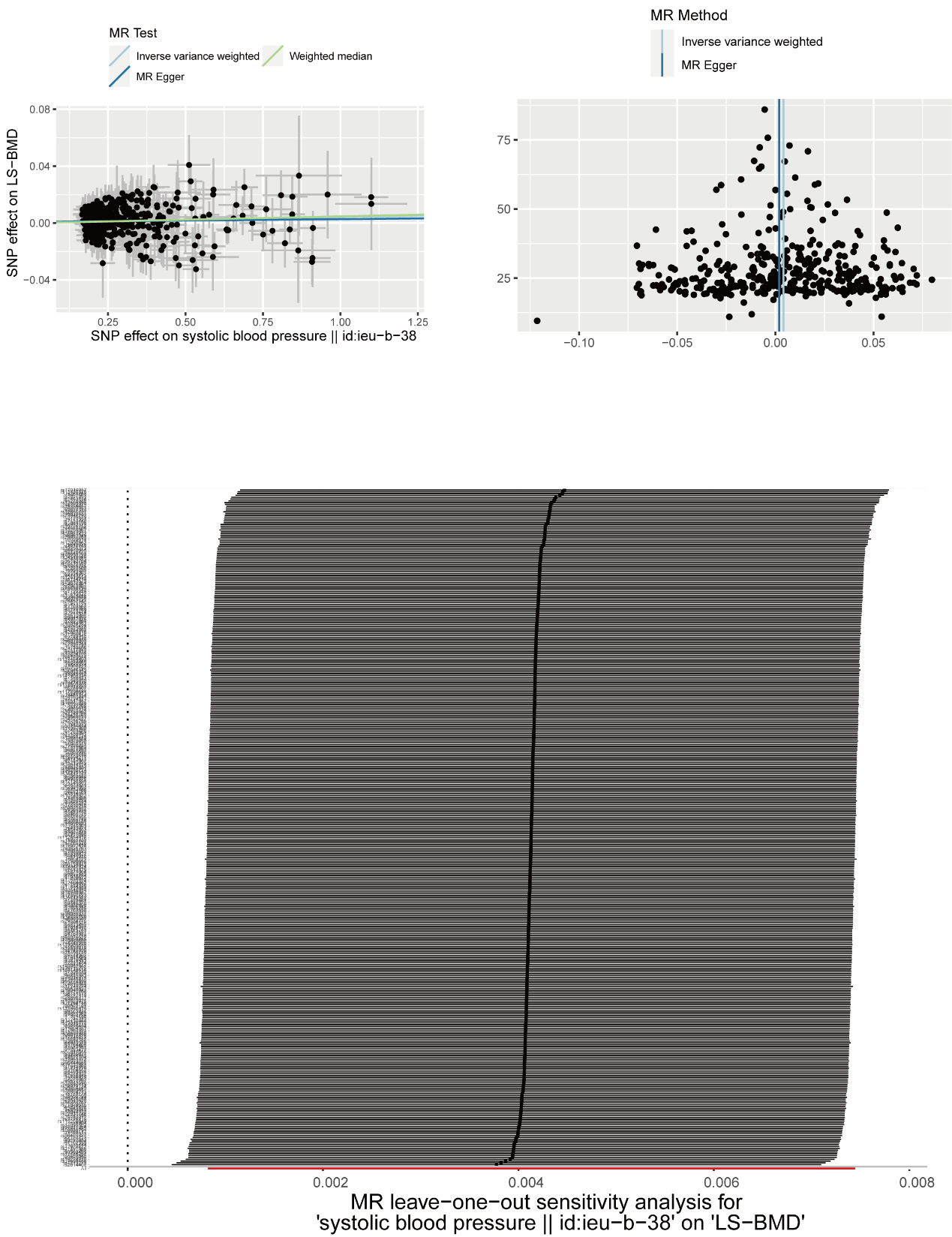


Figure S9 Scatterplot, funnel plot and leave-one-out analysis of relationship between SBP and LS-BMD.


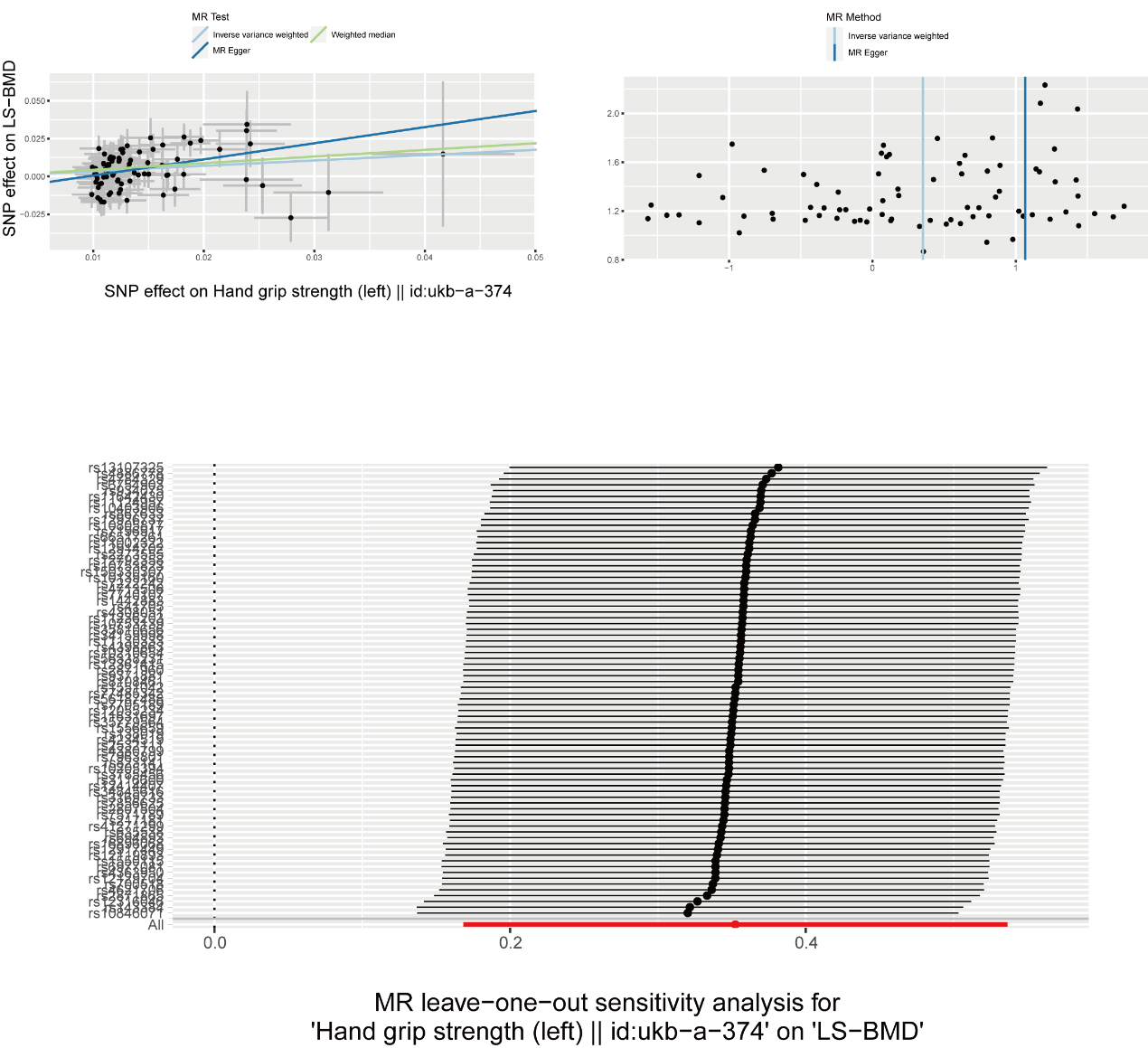


Figure S10 Scatterplot, funnel plot and leave-one-out analysis of relationship between L-HGS and LS-BMD.


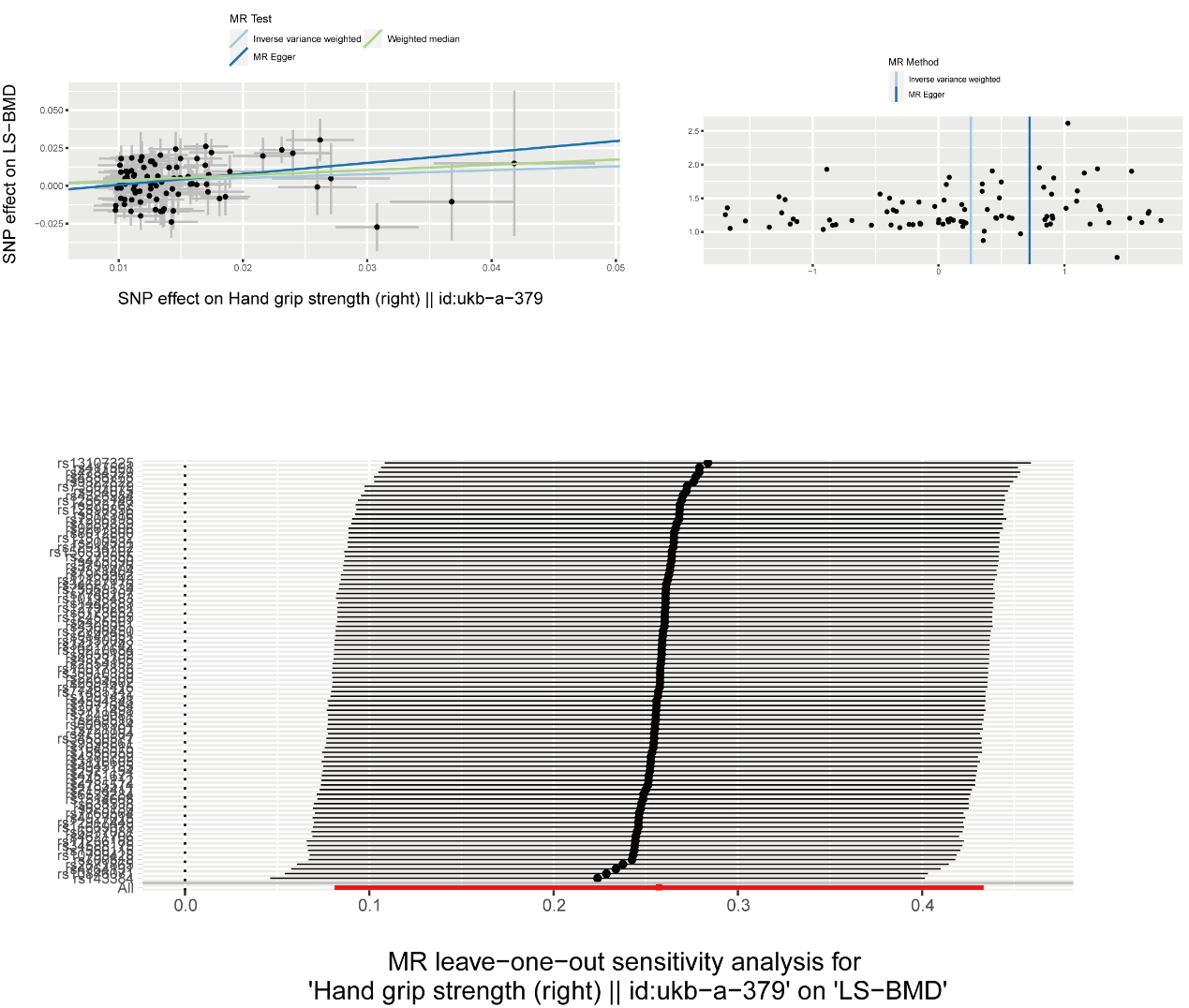


Figure S11 Scatterplot, funnel plot and leave-one-out analysis of relationship between R-HGS and LS-BMD.
